# Fecal-adherent mucus is a non-invasive source of primary human MUC2 for structural and functional characterization in health and disease

**DOI:** 10.1016/j.jbc.2024.105675

**Published:** 2024-01-24

**Authors:** Noah Fancy, Nitin, Darrek Kniffen, Mackenzie Melvin, Negin Kazemian, Javad Sadeghi, Clara A. Letef, Leah D’Aloisio, Amanda G. Copp, Rain Inaba, Geetkamal Hans, Simin Jafaripour, Natasha Haskey, Maitreyi Raman, Pirandis Daneshgar, Kris Chadee, Sanjoy Ghosh, Deanna L. Gibson, Sepideh Pakpour, Wesley Zandberg, Kirk S.B. Bergstrom

**Affiliations:** 1 Biology, University of British Columbia-Okanagan, Kelowna, Canada; 2 Chemistry, University of British Columbia-Okanagan, Kelowna, Canada; 3 School of Engineering, University of British Columbia-Okanagan, Kelowna, Canada; 4 Cumming School of Medicine, University of Calgary, Calgary, Canada

**Keywords:** fecal mucus, host-microbe interactions, inflammatory bowel disease, mass spectrometry, MUC2, mucin-type O-glycosylation

## Abstract

The O-glycoprotein Mucin-2 (MUC2) forms the protective colon mucus layer. While animal models have demonstrated the importance of Muc2, few studies have explored human MUC2 in similar depth. Recent studies have revealed that secreted MUC2 is bound to human feces. We hypothesized human fecal MUC2 (HF-MUC2) was accessible for purification and downstream structural and functional characterization. We tested this *via* histologic and quantitative imaging on human fecal sections; extraction from feces for proteomic and O-glycomic characterization; and functional studies *via* growth and metabolic assays *in vitro*. Quantitative imaging of solid fecal sections showed a continuous mucus layer of varying thickness along human fecal sections with barrier functions intact. Lectin profiling showed HF-MUC2 bound several lectins but was weak to absent for *Ulex europaeus 1* (α1,2 fucose-binding) and *Sambucus nigra* agglutinin (α2,6 sialic acid-binding), and did not have obvious b1/b2 barrier layers. HF-MUC2 separated by electrophoresis showed high molecular weight glycoprotein bands (∼1–2 MDa). Proteomics and Western analysis confirmed the enrichment of MUC2 and potential MUC2-associated proteins in HF-MUC2 extracts. MUC2 O-glycomics revealed diverse fucosylation, moderate sialylation, and little sulfation *versus* porcine colonic MUC2 and murine fecal Muc2. O-glycans were functional and supported the growth of *Bacteroides thetaiotaomicron* (*B.* *theta*) and short-chain fatty acid (SCFA) production *in vitro*. MUC2 could be similarly analyzed from inflammatory bowel disease stools, which displayed an altered glycomic profile and differential growth and SCFA production by *B. theta versus* healthy samples. These studies describe a new non-invasive platform for human MUC2 characterization in health and disease.

The intestinal tract is a biochemically rich reservoir of dietary and microbial lipids, proteins, carbohydrates, and their metabolites, which interface with the host at the mucosal epithelium. The interaction of host mucosa with intestinal luminal content including the microbiota can range from being beneficial to health to becoming deadly due to maladaptive host responses such as chronic inflammation, cancer, and sepsis (1).

Mucins represent a large family of glycoproteins highly expressed at intestinal epithelial surfaces and characterized by domains rich in Serine/Threonine that are sites of glycosidically O-linked oligosaccharide chains (O-glycans) (2). There are approximately 20 members of this family that are broadly characterized as secreted gel-forming (MUC2, MUC5AC, MUC5B, MUC6), secreted non-gel forming (MUC7), and membrane-bound (MUC1, -3A, -3B, −4, −12, −13, −15, −16, −17, −20, & −21) (2, 3, 4). Membrane-bound mucins including MUC1 and MUC4 transduce signals from the environment and influence inflammatory and oncogenic signaling cascades *via* NFκβ and β-catenin activation and modulate inflammatory susceptibility to the microbiota (5, 6, 7, 8). Gel-forming mucins like MUC2 and MUC5AC make gastrointestinal mucus which functions as a barrier to limit exposure to inflammatory microbial pathogen-associated molecular patterns (PAMPS) or toxins, as well as host-derived irritants including proteases and acids (9, 10, 11). Mice lacking the ability to produce gel-forming mucins develop spontaneous chronic colitis and colorectal cancer (9, 12). Due to this, intense research has been directed toward the biochemical characterization of mucus function and its formation. However, technical limitations to mucus study have slowed its progress.

Mucus production is a highly conserved mechanism across animal species to protect mucosal tissues from insult and injury (13, 14, 15). In the colon of humans and many mammals, MUC2 is the primary mucin that makes up the mucus network (16). However, MUC5AC can be expressed during inflammatory conditions to protect against parasite invasion and cytotoxic agents (10, 11, 17, 18, 19). Histologic and *ex vivo* assays of human intestinal tissues demonstrate that MUC2 is specifically and constitutively produced by goblet cells along the entire intestinal tract (20, 21). It is synthesized as a monomer that undergoes extensive posttranslational modification *via* actions of several families of glycosyltransferases (GTs) that first catalyze the condensation of N-acetylgalactosamine (GalNAc) with hydroxyl groups of serine and threonine residues, generating the O-linkage. This initial sugar is extended by other GTs which modify it with various combinations of galactose (Gal), N-acetylglucosamine (GlcNAc), Fucose (Fuc), and Sialic acid (Sia, aka 5-N-acetylneuraminic acid, Neu5Ac). This leads to a diverse repertoire of O-linked oligosaccharides that make up 80% of the mass of MUC2 (1–2 MegaDaltons, MDa) and regulate mucus functions (22). N-glycans also exist on MUC2 which are linked to Asparagine residues and are enriched in GlcNAc and mannose, but these occur at much lower frequency versus O-linked glycans (3, 23). Following these post-translational modifications, MUC2 is extensively polymerized, a feature that allows it, once secreted, to form a formidable glycan-dependent barrier to prevent microbial intrusion into underlying tissues (24, 25, 26). In chronic diseases such as Ulcerative Colitis (UC), a major form of inflammatory bowel disease (IBD), alterations of MUC2 function and glycosylation are well known (26, 27, 28) and animal models have shown definitively that Muc2 and its O-glycans in mice are essential for mucosal barrier function, and protection from microbiota-dependent colitis, colon cancer, and infection (9, 12, 24, 29, 30). This highlights the importance of this molecule to normal physiology and very likely human health.

Despite the abundance of MUC2 in the human colon, there are relatively few studies that have characterized this molecule in depth at the structural and functional level of healthy or diseased individuals. This is because, up until recently, the majority of the unmetabolized barrier-forming mucus was thought to be firmly adherent to the epithelial cells of the colonic mucosa (31). As a result, mucus was perceived to be accessible under limited and highly regulated ethical constraints, including colon biopsies (26), surgeries, or access to cadavers (32). Even under these conditions, there is no guarantee of “normal” mucus: Colon surgeries or resections usually happen under disease conditions; colons harvested from a deceased individual likely have many variables at play (time, terminated gut physiology, abnormal gut microbial activities, the release of lysosomal glycosidases from dead cells into the gut) that can influence the colon mucus phenotype in ways that are not yet understood. Thus, the amount of primary human MUC2 obtained by these approaches is either very little (*e.g*., biopsies), or even when abundant, may also be compromised given the context in which it is acquired (*i.e*. diseased or dead tissues).

As a result, many high-profile studies addressing the functional capacity of mammalian mucins have relied on animal sources of mucin, including commercial preparations of porcine gastric mucin (33), or porcine colon mucin obtained from farm animals (34). These studies have led to some major insights into the biological functions of glycans in relation to commensal (34) and pathogenic responses (35, 36). However, because these mucins are either from a different tissue or different species, questions remain about whether human colonic MUC2 will have a differential impact relative to the non-human Muc2 that is analyzed. Thus, there is a need to identify means to have more ready access to human MUC2 for structural and functional studies and to characterize the glycome in larger populations.

Recently, our understanding of the gut mucus network has advanced. Based on a group of studies in animals as well as humans, it has been revealed that much of the normal mucus structure is not firmly bound to the mucosal wall but instead is bound to feces (37, 38, 39). Indeed, we have shown that a thick and continuous mucus barrier layer bound to the fecal surface retained the established bacterial impenetrable abilities of mucus (37). We therefore reasoned that, since mucus is readily harvestable from gut mucosal scrapings for analysis, we could do the same to exploit the fecal adherent mucus as a previously untapped source of primary human MUC2 for structural and functional characterization. Our results show for the first time that human fecal mucus can be readily harvested from human fecal material and analyzed simultaneously at the structural, glycomic, and functional levels. These studies open the door to many new opportunities for researchers to understand this essential molecule, how it compares among healthy populations, and how it changes in disease.

## Results

### Human and mouse fecal-adherent mucus shows structural similarity *in situ*

**Figure 1:**
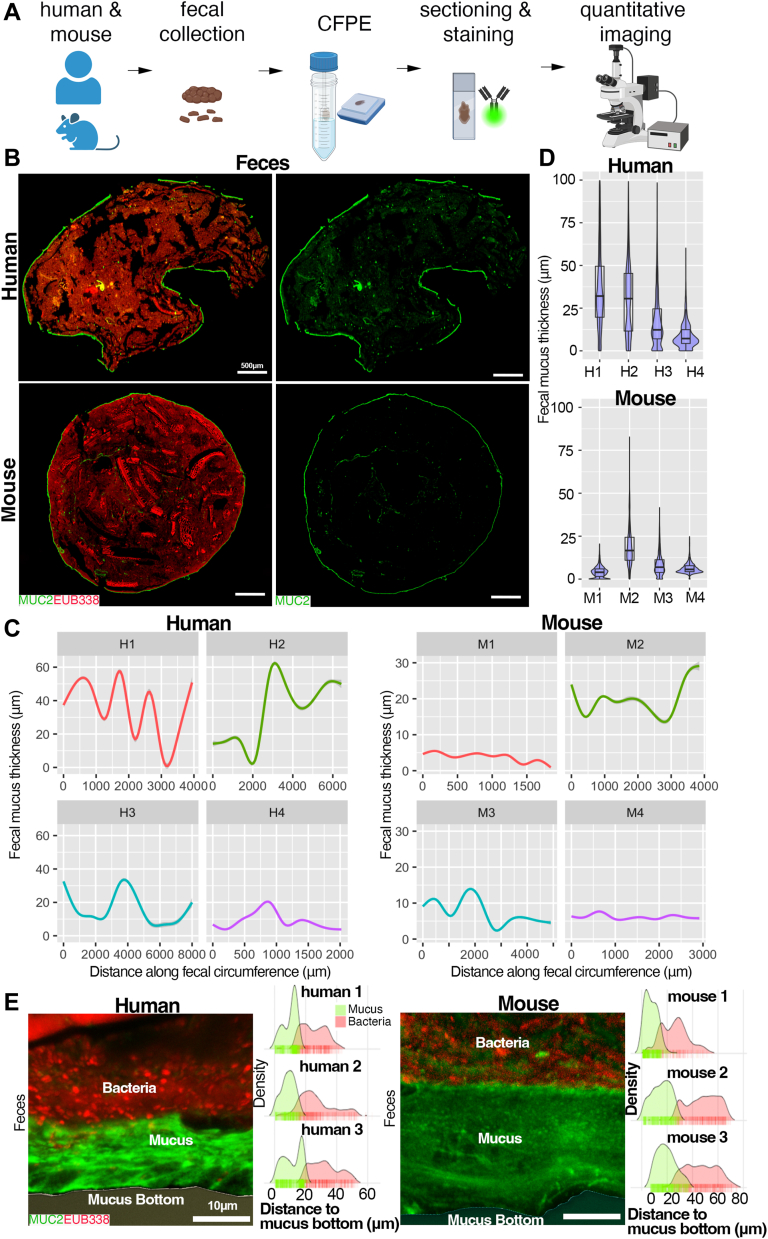
**Visualizing and quantifying mucus barrier functions on fecal sections.***A*, processing scheme for feces for mucus analysis *in situ*. *B*, tiled cross-section of healthy human feces (*upper*) and mouse feces (*lower*) dual stained with FISH probes (*red*) and a rabbit polyclonal antibody targeting human MUC2 (see Experimental procedures; *green*). *C,* quantitative imaging of mucus thickness along the distance of fecal periphery. *D*, Violin plot of total mucus thickness of four independent human and mouse samples. *E*, confocal imaging of MUC2:FISH-stained sections. *Right of image*: Density plots of mucus thickness and microbial distance to mucus bottom showing microbial penetration into the mucus.

To validate previous studies (37), and further explore the analytical capabilities of fecal mucus, we first took surface slices (1–2 mm thick) from healthy adult fecal donors as well as feces from wild-type (WT) mice as a control, fixed them in mucus-preserving Carnoy’s fixative (40), processed them for embedding, and sectioned them for histochemical staining (Fig. 1*A*). We stained these samples for MUC2 using a rabbit polyclonal anti-MUC2 IgG antisera originally developed against the colon carcinoma line LS174T (41), as well as the bacterial microbiota using a universal bacterial-specific probe (EUB338) for fluorescence *in situ* hybridization (FISH) (42), and visualized the mucus structure and microbiota by fluorescence microscopy (Fig. 1*B*). Aside from mechanical breaks due to sectioning the fibrous fecal mass, we found a clear continuous mucus layer adherent to the fecal mass that was encapsulating the community in both humans and mice (Fig. 1*B*). Using computational approaches, we performed quantitative imaging of thickness along a distance of multiple fecal boli (see Methods) from a south-Asian female (“H1”), a south-Asian male (“H2”), a Caucasian female (“H3”), and a Caucasian male (“H4”), to get a sense of mucus continuity and thickness variation across large (mm) distances at high resolution (over 12,000–20,000 points along the layer) (Figs. 1*C* and S1, *A*–*D*). We saw the mucus coating was continuous across the fecal bolus, although thickness varied substantially along these distances in the four samples analyzed (Fig. 1*C*), from ∼5 μm –60 μm thick. A similar variation could be seen with mouse fecal mucus encapsulation (Figs. 1*C* and S2*A*). Notably, a plot of median thickness shows that overall thickness was different across samples, highlighting the dynamic nature of this layer (Fig. 1*D*). However, the mean thickness between human samples (18.9 ± 14.4 μm, n = 4) and mice (9.97 ± 6.22 μm, n = 4) was not statistically significant (*p* = 0.3034, Welch two-sample *t* test)), although this may reflect the low sample size. High magnification confocal imaging showed a clear barrier capacity of the mucus layer, with most microbes not penetrating the mucus network, consistent with the known functional roles in rodent systems (Fig. 1*E*). These studies confirm previous work showing a robust mobile human mucus barrier layer attached to feces (37).

Because these samples were “idealized” in that they were fresh and solid, we explored whether an intact analysis could be performed on samples that were previously frozen and then fixed, as well as on samples that were not solid. We found that by taking fecal surface slices from samples stored at −20 °C, and then fixing these samples directly in Carnoy’s solution and processing as above, an intact mucus barrier could be visualized and measured (Figs. 1*C*, “H3”, and S1*C*). Further, we found that loose stools rarely had a continuous layer, but instead showed abundant mucus within the fecal mass (Fig. S2*B*), consistent with previous work (43). Staining with an irrelevant rabbit IgG isotype and nonsense FISH probe (NON338) revealed the specificity of the MUC2 and microbial labeling (Fig. S2*C*). These studies indicate that structural analysis of the encapsulating adherent mucus can use fresh or frozen stools, but is most easily analyzed on firm stools.

### Fecal mucus can be extracted and purified from human fecal material for proteomics

**Figure 2:**
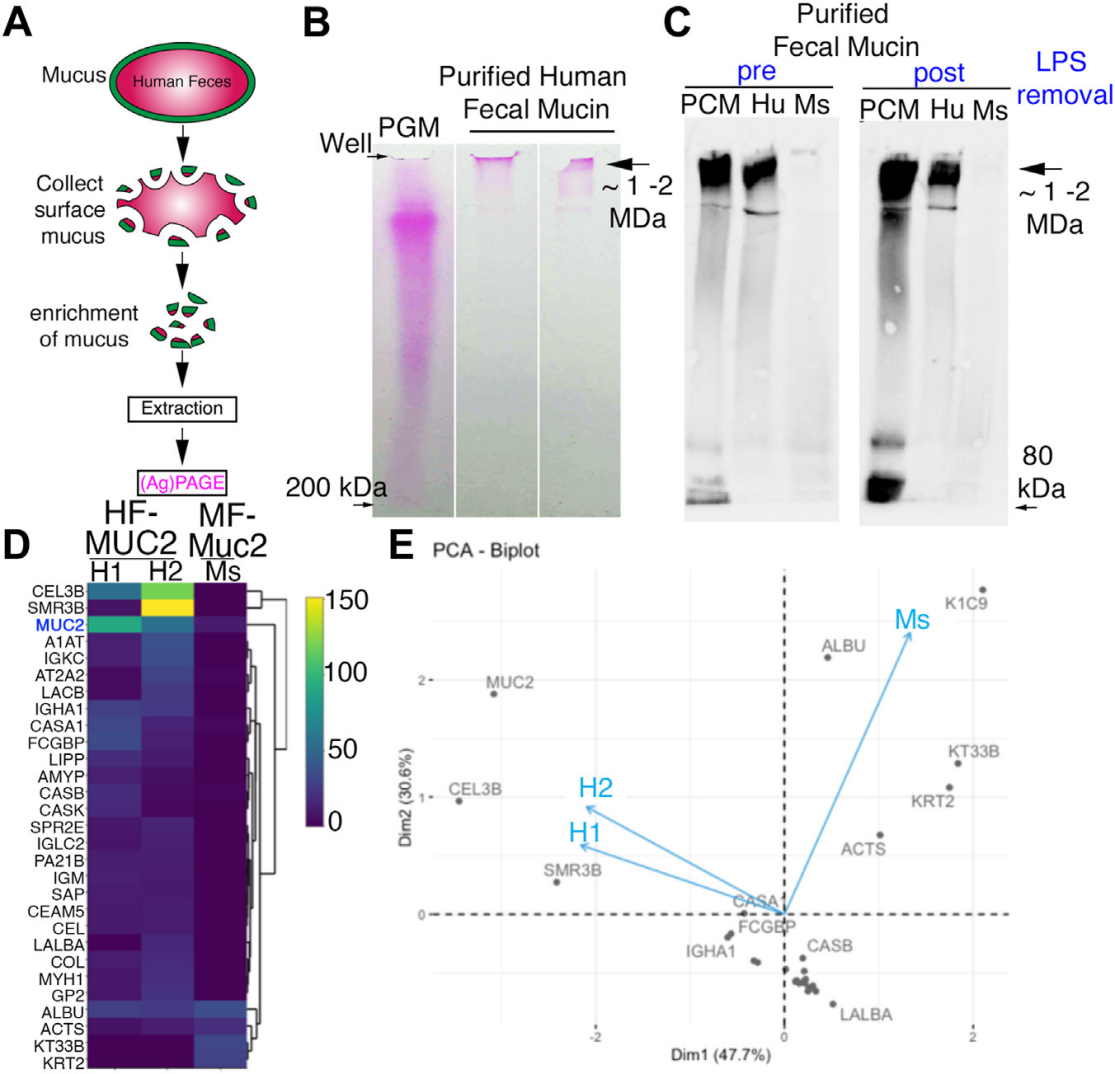
**Confirmation of MUC2 extraction from feces**. *A*, schematic of human MUC2 extraction and purification. *B*, periodic Acid-Schiff staining of mucin-purified from feces. PGM = commercial crude porcine gastric mucin as a positive control for high molecular weight mucin. *C*, Western blot of MUC2 (rabbit polyclonal IgG, used in Fig. 1) purified from feces and separated by SDS-UAgPAGE pre- and post-LPS removal. Arrow indicates high molecular weight MUC2. White space between lanes indicates these lanes were not adjacent on original gel. PCM, Porcine colonic mucin; Hu, human; Mouse, m . *D*, Heatmap of spectral counts of proteomic profile from extracted MUC2/Muc2 from human and mouse. *E*. PCoA biplot of proteins found on human MUC2.

Given our observation of intact mucus on human feces, and our previous findings showing the encapsulating layer surrounding fecal material can be extracted and analyzed biochemically in mice (37), we reasoned this should also be possible with human fecal-adherent mucus. To do this, we took surface slices that were enriched for the human fecal MUC2 (HF-MUC2)-rich mucus (based on immuno- and epifluorescent staining above) and extracted the guanidium chloride (GuCl)-insoluble MUC2 polymer using previously-established approaches for murine colonic mucus (44, 45). To this end, the fecal mucus was extracted, reduced, alkylated and then dialyzed into water, and run through an endotoxin removal kit to remove potential contaminating endotoxin (lipopolysaccharide, LPS) (Fig. 2*A*, see also Experimental procedures). Notably, prior to LPS removal, we also noticed an off-white precipitate in the dialyzed mucins that was removable *via* centrifugation or endotoxin removal, suggesting it was a complex of LPS (Fig. S3*A*). Post-LPS removal, we were able to acquire 5.38 mg from 5 g of starting material. We separated the purified HF-MUC2 extraction electrophoretically along with a commercial mucin prep (porcine gastric mucin, PGM) as a size calibrator (ranges up to >1 Mega-Dalton, MDa) and visualized the mucins by in-gel periodic acid-Schiff (PAS) staining. We noted high molecular weight PAS-positive bands, ∼2 MDa in size, in the human fecal mucus samples that ran at rates even slower than PGM (Fig. 2*B*), suggesting successful extraction of the oligosaccharide-rich MUC2. Following separation *via* composite SDS-Urea-Agarose-Polyacrylamide gel electrophoresis (SDS-UAgPAGE), Western analysis using the rabbit polyclonal anti-MUC2 antibody (used for *in situ* labeling (Fig. 1)) revealed a positive signal in the high high molecular weight regions, confirming the presence of MUC2 in these human fecal samples (Fig. 2*C*). This MUC2 antibody also bound strongly to porcine colonic mucin and weakly to mouse fecal colonic mucin (Fig. 2*C*). Notably, similar levels of HF-MUC2 were observed in the LPS-rich precipitate compared to soluble MUC2 in the supernatant, suggesting that: (i) the precipitate is a complex of LPS and MUC2 (Fig. S3*B*), and (ii) LPS removal is necessary for maximum recovery of soluble MUC2 from fecal preparations. We also extracted mucus from the loose fecal material, finding that while the yield was lower, intact mucus was still obtained (Fig. S3*C*); this was consistent with our in-situ observations of broken mucus within the loose material (Fig. S1*D*).

To confirm HF-MUC2 presence in these extracts by an independent method, we conducted a proteomic analysis of the human fecal mucin extract and ran it against murine fecal mucin (Fig. 2, *D* and *E*). Notably, the top hits for proteomics included known contaminants in proteomic studies including trypsin (an artifact of proteomic processing) (46) and various type 1 and type II Keratins (K2C1, K1C10, and K1C9) which are widely considered to be introduced *via* sample handling (47, 48) and albumin. Given these human proteins were also observed in the mouse MUC2, we concluded they represented contaminants introduced at an unknown point post-dialysis since these molecules are under 100 kDa, the molecular weight cut-off point during dialysis. After correcting for these contaminants, the results show MUC2 (but no other MUC family member) to be among the most abundant proteins in one prep; however, others that co-purified at a similar level were Chymotrypsin-like Elastase 3B, normally expressed in pancreatic secretions (49), and SMR3B (submaxillary gland protease 3B), a protein distantly related to mucins (50) (Fig. 2*D*). We noted several other proteins at low abundance including Fc gamma-binding protein (FCGP), well-known bind to and likely stabilize MUC2 (51), indicating our purification procedure can capture HF-MUC2-binding proteins as well as HF-MUC2 (Fig. 2*D*). Collectively, these studies show human colonic MUC2 can be extracted from feces and biochemically analyzed.

### Characterization of human fecal MUC2 O-glycosylation *in situ*

**Figure 3:**
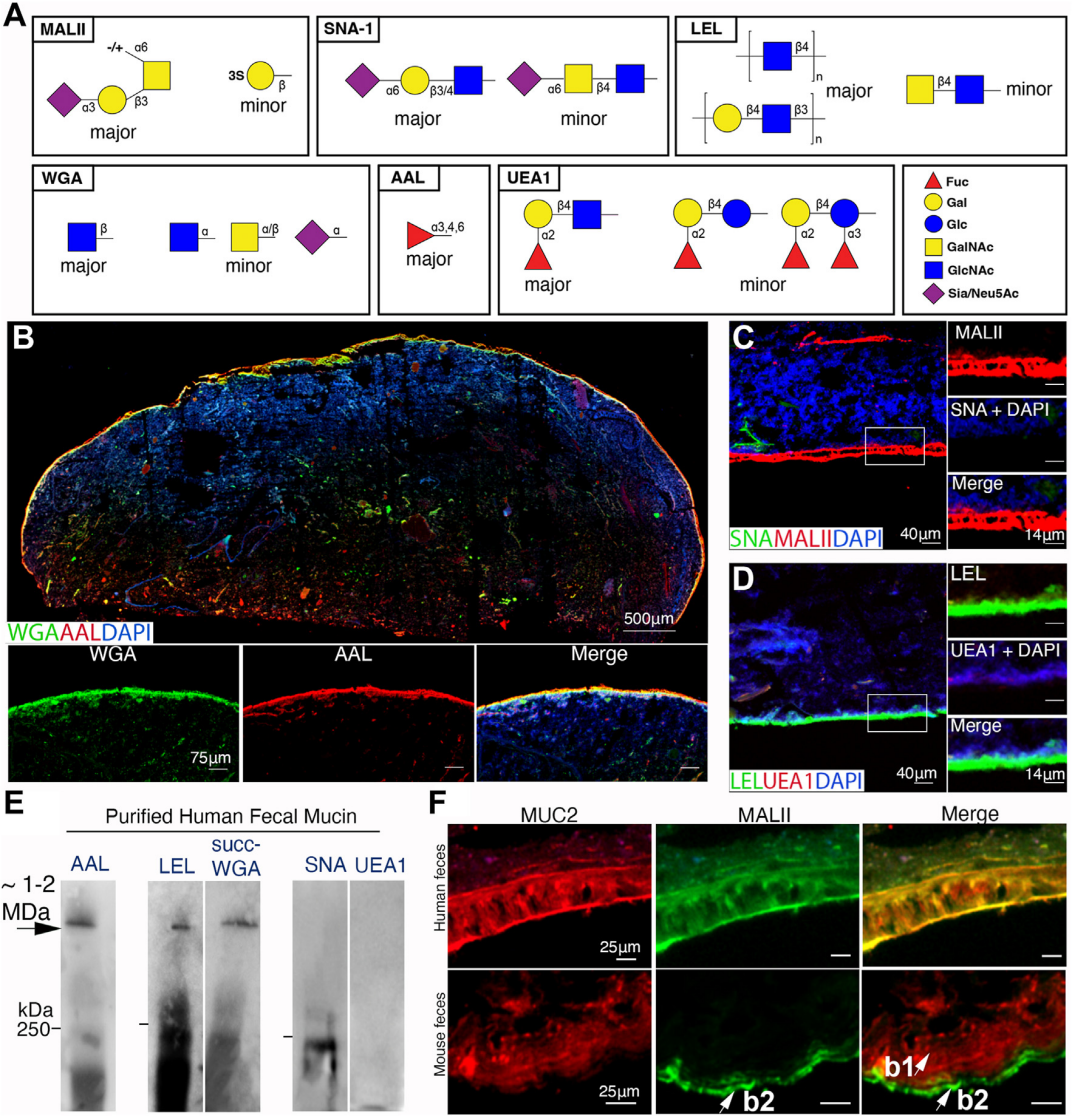
**Analysis of MUC2 glycosylation on fecal sections *in situ***. *A*, schematic of glycan structures recognized by the lectins used in this figure to assist in interpretation. *B*, Tiled cross-section of healthy human feces dual stained with Wheat Germ Agglutinin (WGA) and *Aleuria aurantia* lectin (AAL). *C* and *D*, high-mag imaging human mucus stained for *Maackia ammurensis* (MAL)-II and *Sambucus nigra* (SNA)-1 (*B*), and *Lycopersicon esculentum* (Tomato) lectin (LEL) and *Ulex europaeus*-1 (UEA-1) (*C*). *E*, Lectin blotting of fecal MUC2. Lanes that are close together represent samples run on the same gel and transferred to the same membrane, but separated for specific blots. White space indicates lanes were not adjacent. Bar on the right of blot = 250 kDa maker. *F*, dual stain for MUC2 and MALII to analyze MALII distribution in human versus mouse fecal sections. Results are representative of five individual fecal sections.

MUC2 is hyper O-glycosylated (at least 200 O-glycosides per monomer (52)), which is important to characterize due to the influences of O-linked oligosaccharides on the structural and functional properties of MUC2 and the mucus gel (22). However, there are relatively few studies that have explored this at a visual or biochemical level. Furthermore, glycan profiling on CFPE sections can provide novel insights on mucus organization *in situ*, as demonstrated in mice (37). Given the fidelity of the MUC2 mucus network on human feces, we first probed human fecal sections using a panel of lectins (Fig. 3*A*). We started with Wheat Germ Agglutinin (WGA), which broadly targets sialylated and GlcNAc-ylated epitopes on glycoconjugates including mucin (53), as well as *Aleuria aurantia* lectin (AAL) which targets α1,3/4-linked Fucose (Fuc) on N- and O-glycans, and Fucα1-6 GlcNAc on N-glycan cores (54, 55). We found fecal mucus stained strongly for both these lectins, indicating the glycosylation of MUC2 on human sections is well preserved by our methods (Fig. 3*B*). We noted a reduction but not complete ablation of the WGA signal upon sialidase treatment in mouse and human samples, suggesting both sialylated and GlcNAc-ylated glycans are present on MUC2 (Fig. S4*A*), or that desialylation was not complete.

To further characterize sialylation and fucosylation, we performed further combinations of lectins. For sialylation, we probed the tissues with *Maackia amurensis* agglutinin (MALII) which binds Sia(α2-3)Gal or 3-O-sulfated Gal (56) in combination with *Sambucus nigra* lectin (SNA1), which binds Sia(α2-6)Gal (57). We found a strong signal for MALII, but surprisingly no signal for SNA1 in most human samples (n = 5) (Fig. 3*C*). We found the treatment of human fecal sections with neuraminidases targeting α2,3,-6, or −8 linked Sia did not impact staining with MALII (Fig. S4*A*), suggesting the MALII ligand is sulfated in humans similarly to mice (37). The SNA absence was unlikely due to O-acetylation which can block SNA binding (58), as de-O-acetylation *via* NaOH treatment did not increase the SNA signal on human MUC2, despite it doing so on mouse goblet cells and mouse lamina propria (Fig. S4*B*). Of note, NaOH treatment ostensibly unmasked MALII binding α2-3 sialylated epitopes on lamina propria cells, confirming the ability of this lectin to recognize this linkage *in situ* in our hands (Fig. S4*B*). For fucosylation, we focused on an important epitope, Fucα1,2Gal, which is recognized by *Ulex europaeus* agglutinin I (UEA1) (57). This epitope is the product of the Secretor gene *FUT2* (59) and is found on O- and N-glycans. Surprisingly, human MUC2 was negative for UEA1, although it was positive for *Lycopersicon esculentum* lectin (LEL) which binds [Galβ1,4GlcNAcβ1,3]n indicating the presence of type II polylactosamine on N- and O-glycans (57) (Fig. 3*D*), and suggesting neither glycan class could be labeled by UEA1. We addressed the possibility that O-acetylation and sialyation could block UEA1 binding to human MUC2 (60), but we did not observe any increase in signal with this treatment (Fig. S4*C*). Western analysis agreed with the in-situ results (Fig. 3*E*).

The mucus barrier layer in mice is a composite of a major b1 layer and a minor b2 layer (37), but whether humans have a similar organization is unknown. We therefore analyzed this directly by comparing fecal mucus structure from humans and mice *via* staining them for MUC2, and MALII, a combination which reveals the b1 barrier layer (MUC2^+^MALII^-^) and b2 barrier layer (MUC2^+^MALII^+^) in mice (37). Interestingly, while the b1/b2 barrier layers were readily observed in mice as expected, we did not observe this in human MUC2; in fact, the MALII^+^ layer was in what would be equivalent to the b1 layer (Fig. 3*F*). This was also supported by confocal analysis of MALII/MUC2 stained sections, where MALII colocalized with MUC2, indicating also the targeting of the lectin to MUC2 (Fig. S4*D*). These results point to some major differences in glycosylation and mucus barrier substructure of human fecal MUC2 *versus* mice, consistent with previous work (61), as well as how mucus is structurally organized in humans. Overall, these studies indicate human MUC2 glycosylation can be readily analyzed *in situ*, *via* western blotting, and this information can be used to compare the mucus network structure between species.

### Cross-species comparison of human fecal Mucin-2 O-glycosylation

**Figure 4:**
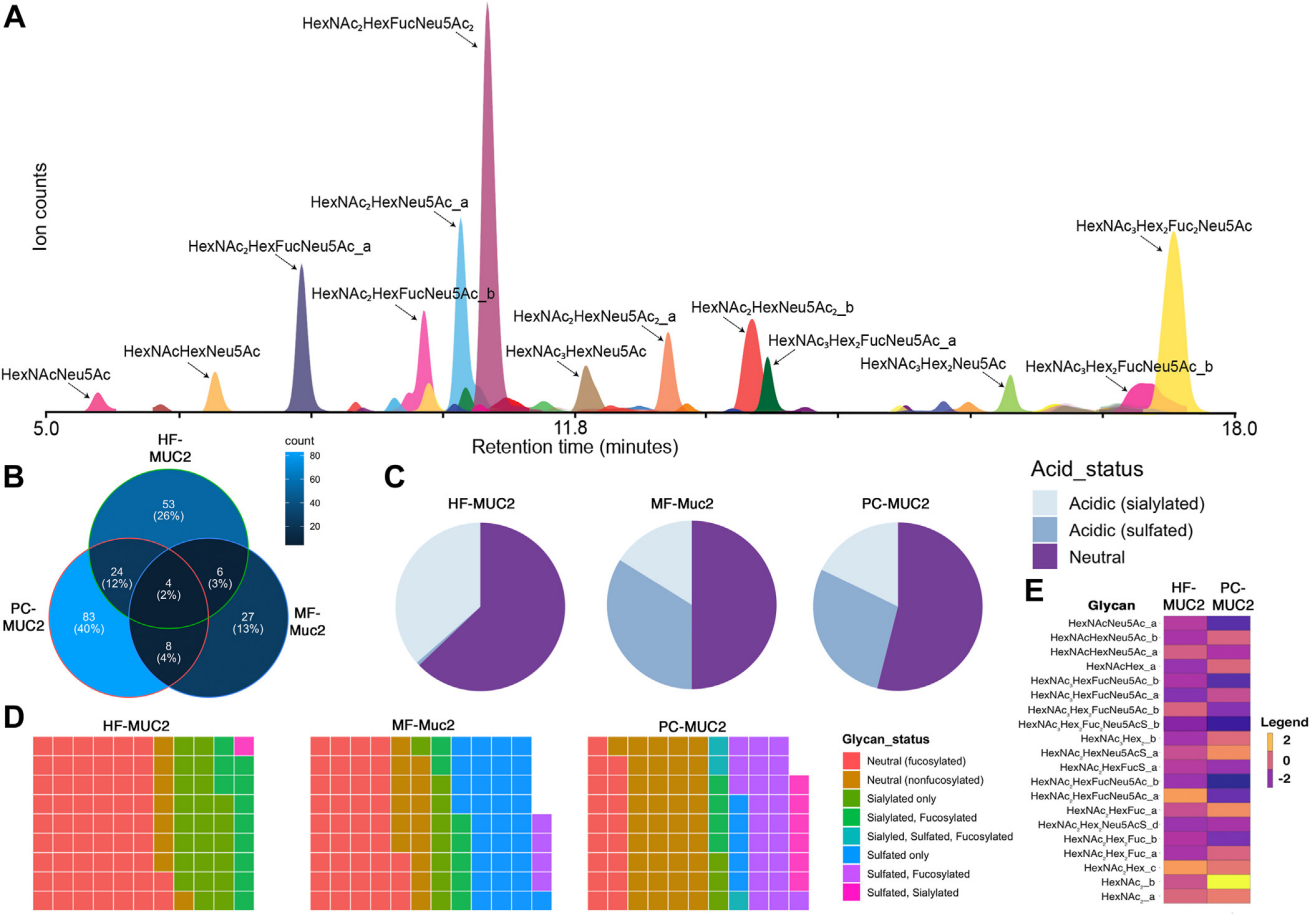
**Comparative glycomic analysis of human fecal MUC2 with other sources**. *A*, representative extracted ion chromatograms (EICs) of all sialylated O-glycans detected after HPLC-MS analysis of a single human sample. Each peak represents a sialylated glycan. Not all peaks are annotated for simplicity. Annotations refer to monosaccharide compositions (HexNAc = N-acetylhexosamine; Hex = Hexose; Fuc = Fucose, Neu5Ac = Sialic Acid; subscripts = number of monosaccharides. The peak areas for all glycans were normalized to the totals for the remaining comparisons in Panels b through e. *B*, Venn diagram showing overlap of common glycan structures identified from ammonia-catalyzed beta-elimination of O-linked oligosaccharides from indicated sources. Total unique and common glycans are indicated as total number with % of total analyzed in parentheses. *C*, pie chart of overall acidic *versus* neutral glycans from human fecal, mouse fecal and porcine colonic MUC2. *D*, waffle plot showing relative abundance of specific classes of glycans indicated by color. *E*, Heatmap of log2-transformed relative abundances of individual glycans found in both human and porcine colon mucin sample, with each row representing a unique structure. HF-MUC2, human fecal MUC2; MF-Muc2, mouse fecal Muc2; PC-MUC2, porcine colonic MUC2.

Many informative studies use porcine colonic mucin (PCM), obtained from pig colon mucosal scrapings to investigate MUC2 oligosaccharide interactions with human symbionts (34, 36). However, it is unclear how closely PCM resembles human MUC2, especially the fecal-associated, which is what the microbes are interacting with at a given time. Given the differences observed with mouse MUC2, we designed a study to compare head-to-head human fecal MUC2 (HF-MUC2) with porcine colonic MUC2 (PC-MUC2), and mouse fecal Muc2 (MF-Muc2). We therefore extracted MUC2/Muc2 from human and mouse feces, as well as pig colon scrapings, and then obtained complete glycomes of the glycan alditols yielded upon reductive β-elimination and analyzed them by HPLC-QToF-MS using basic, ammonium formate-containing mobile phase well-suited for the detection of acidic (sialylated or sulfated) structures (62, 63). A representative extracted ion chromatogram (IEC) displaying all of the sialylated glycans detected in an HF-MUC2 sample is depicted in Figure 4*A*; peak areas for all putative glycans detected by HPLC-MS were acquired and normalized to the total glycome in each sample to permit semi-quantitative comparisons. Because of the luminal presence of the mucins, there is potential for O-glycan degradation in the extracted MUC2. Therefore, we assessed whether the HF-MUC2 and MF-Muc2 had “intact” glycoprofiles by comparing the overall length of the oligosaccharides identified by MS between the fecal and the relatively “unmetabolized” PC-MUC2 (Fig. S5*A*). We found that fecal MUC2 displayed a similar frequency of glycans that were >5 monosaccharides long compared to PC-MUC2 and MF-Muc2. Only three glycans in PC-MUC2 were greater in size than in human MUC2 (Fig. S5, *A* and *B*). Further, if extensive degradation was occurring before extraction, we would predict a strong negative correlation between glycan size and relative abundance (relative abundance increases as glycan size decreases) in the fecal-extracted MUC2 but not tissue-extracted MUC2 (PCM). However, there was no correlation between glycan length and the relative abundance of individual glycans (Fig. S5*B*). These studies suggest fecal-adherent MUC2 harbors an overall unmetabolized O-glycome.

We next looked at how much overlap exists in the O-glycan profiles between the three species. As depicted in the Venn diagram (Fig. 4*B*), there was 9% overlap between glycomes from HF-MUC2 and PC-MUC2, 6% overlap between HF-MUC2 and MF-Muc2, and remarkably only 2% of glycan structures detected were seen in common between all species.

Upon analysis of overall acidity based on sialylated or sulfated structures, we observed in this group that PC-MUC2 and MF-Muc2 had an overall higher proportion of acidic glycans (40–50%), which was due to both sialylated and sulfated structures. However, HF-MUC2 showed an overall lower level of acidic glycans (30%), due to minimal sulfation *versus* the other two species indicating the level of sialylated-only (*i.e.* non-fucosylated or non-sulfated) glycans was greater in humans *versus* the other species (Fig. 4*C*) in this proof-of-principle comparison. To get a higher resolution of the difference between glycan groups, we stratified the glycomes based on fucosylation status, neutrality, sialylation or sulfation with or without fucose or sialic acid. Strikingly, compared to PC-MUC2, HF-MUC2 had fewer neutral nonfucosylated (*i.e.* likely I antigen) and a higher proportion of glycans containing just Hex, HexNAc and Fuc (likely blood group H or Lewis structures) (Fig. 4*D*). In addition, fucosylated glycans were preferentially sialylated on HF-MUC2 but were sulfated on PC-MUC2 (Fig. 4*D*). There were also more glycans with both sialylated and sulfated structures in PC-MUC2 *versus* HF-MUC2 (Fig. 4*D*). Notably, most sulfated structures were non-fucosylated or non-sialylated in MF-Muc2 (Fig. 4*D*). N-glycolylneuraminic acid (Neu5Gc) is a product of the CMP-N-acetylneuraminic acid hydroxylase (CMAH) which is absent in humans (64). Thus, the only source of Neu5Gc would have to come from the diet (*e.g.*, pork), followed by its salvaging by goblet cells and incorporation into glycosylation pathways in the Golgi (65). We did not find a single Neu5Gc^+^ glycan in HF-MUC2, or MF-Muc2, although several glycans in PC-MUC2 unambiguously demonstrated the presence of this sugar (*e.g.*, HexNAc₂Neu5Gc_b) (Fig. S5*C*). The studies suggest a dietary source of Neu5Gc was not likely incorporated into the MUC2 O-glycome in this particular human sample.

To observe if any unique glycans were contributing to these differences, we analyzed the relative abundances of individual glycans. Surprisingly, each species had unique glycans that contributed disproportionally to the overall fecal MUC2 O-glycome (Fig. S5*D*): HF-MUC2 had a tetrasaccharide composed of HexNAcHex_2_Fuc_1_ that contributed up to 51.1 ± 6.2% (n = 3 technical replicates) of the relative levels of glycans, potentially explaining the high Fuc levels in the HF-MUC2 sample. In PC-MUC2, a disaccharide of two HexNAcs, likely a core 3 (GlcNAcβ1,3GalNAc) structure, made up 22.8 ± 2.8% of total glycans, potentially explaining the larger proportion of neutral non-fucosylated glycans *versus* the other species' samples. In MF-Muc2, a sulfated hexasaccharide composed of HexNAc_3_Hex_2_S_1_, contributed to 28.8 ± 8.2%, which could explain the larger sulfated (non-fucosylated/sialylated) glycan representation in MF-Muc2. Removing these high-representation glycans did not affect the correlation analysis (Fig. S5*E*).

Finally, we examined the relative abundances of the glycan structures that were common between HF-MUC2 and PC-MUC2. Of the common structures, most contributed to a small proportion of the overall O-glycome (relative abundances ranged from ∼0.02–4%. (Fig. 4*E*). However, the largest differences emanated from the HexNAc_2_ disaccharide, present in both species with equal retention times, but was only 0.55% in HF-MUC2, ∼45-fold lower than in PC- MUC2. (Fig. 4*E*). Collectively, these studies point to how HF-MUC2 harbors an intact glycome that can be used to compare glycosylation between samples. In this case, we could demonstrate that glycan diversity is driven by fucosylation and sialylation status in HF-MUC2; by sulfation, fucosylation, and sialylation in PC-MUC2, and primarily by sulfation and fucosylation in MF-Muc2.

### *In-situ* investigation of MUC2 in healthy *versus* IBD samples

**Figure 5:**
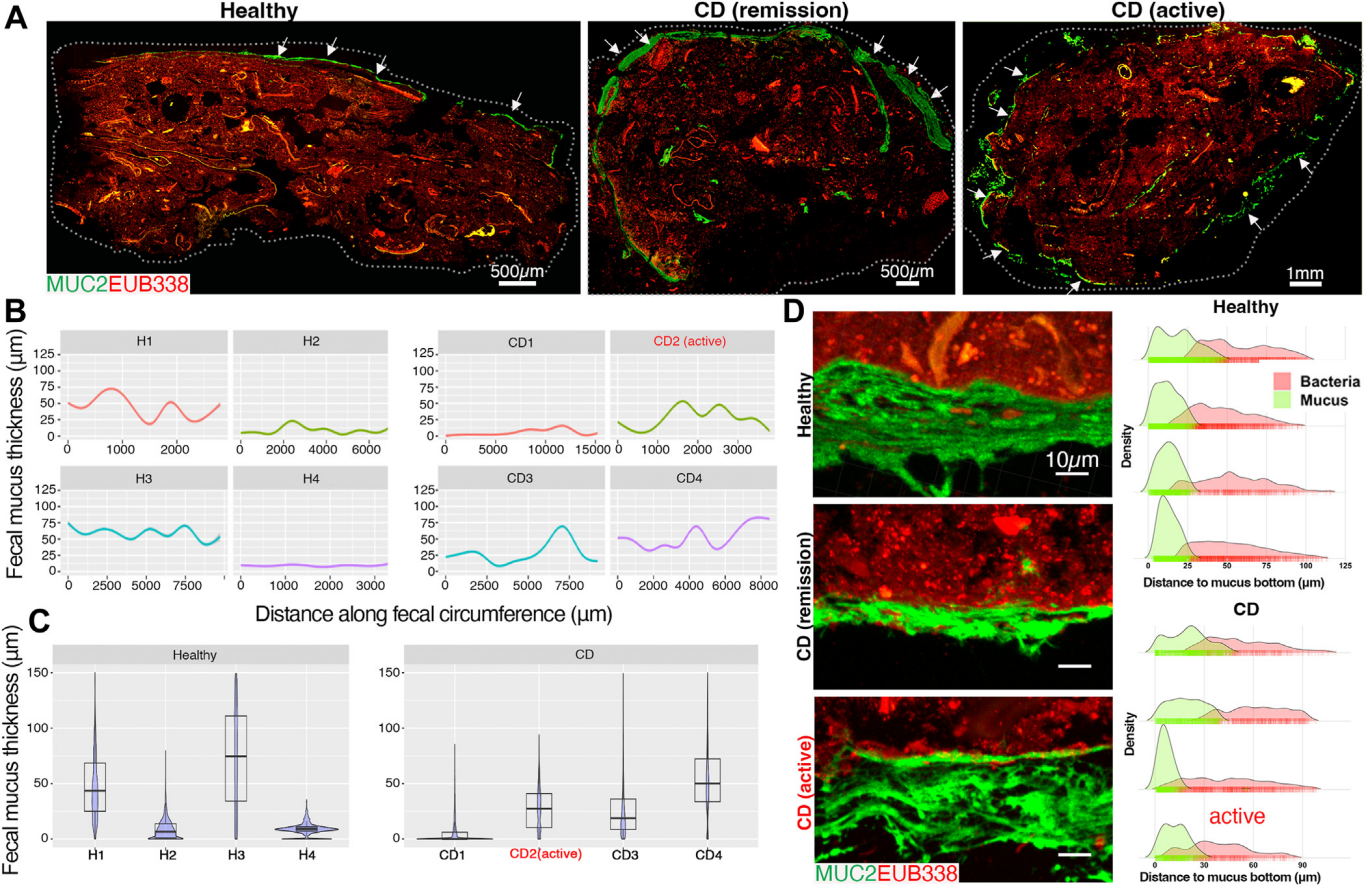
**Comparison of human fecal MUC2 from healthy and Crohn’s Disease samples**. *A*, tiled cross-section of human feces from healthy persons or Crohn’s Disease (CD) patients dual-stained with FISH probes (*red*) and an AF488-conjugated mouse monoclonal antibody targeting human MUC2 (see Methods; *green*). The dotted line denotes the boundary of the image. *B*, quantitative imaging of mucus thickness along distance of fecal periphery. *C*, Violin plot of total mucus thickness of four independent healthy and CD samples. *D*, confocal imaging of MUC2:FISH-stained sections. *Right of image*: Density plots of mucus thickness and microbial distance to mucus bottom showing microbial penetration into the mucus.

The above analysis led us to investigate whether we could compare mucus structure between healthy and disease conditions. Inflammatory bowel disease (IBD) represents a chronic relapsing/remitting inflammatory disease of the gastrointestinal tract previously shown to exhibit altered O-glycosylation profiles on tissue-extracted MUC2 (27). IBD includes the major categories of Crohn’s Disease (CD) and Ulcerative Colitis (UC). We were able to acquire a fresh stool sample set from adult CD patients (including states of moderate active disease and remission) and compare them with healthy samples. We first conducted an *in situ* investigation on mucus thickness and quality on CFPE sections. Both healthy (n = 4) and CD (n = 4) stool exhibited a clear MUC2^+^ adherent mucus along the periphery (Fig. 5*A*), with mucus structure varying in thickness along the distance within a sample, and varying widely in thickness between samples (Figs. 5*B* and *C* and S6*A*). A disrupted structure was observed in the active CD sample (solid stools), while we observed a thick continuous mucus in a patient in remission (solid stools) *versus* healthy control (Figs. 5, *A*–*C*, and S6*A*). Notably, confocal analysis of bacterial MUC2 interactions revealed that in this small cohort, microbes were found deeper in the MUC2 barrier layer in active disease and remission CD HF-MUC2 samples (Fig. 5*D*). Because some of the CD samples were from moderate disease, it remained possible that other secreted mucins including MUC5AC and MUC4 could be observed in the mucins as they have been previously reported to have increased colonic expression in humans and murine models (11, 18, 66, 67). We therefore stained healthy and CD fecal sections with antibodies targeting MUC5AC and MUC4, finding all samples were negative for these mucins (Fig. S6, *B* and *C*). While we cannot make any major conclusions between groups with our current metrics due to our smaller sample size, these studies demonstrate that MUC2 functions can be analyzed *in situ* in a chronic disease setting.

Lectin analysis revealed both healthy and CD HF-MUC2 to be positive for AAL (fucosylated), MALII (α2,3 sialyated or 3-O-sulfated), and LEL (Type II LacNAc), and absent for UEA1 (α1,2-Fuc). Notably, we noted SNA (Neu5Acα2,6Gal) on MUC2 was more readily observed in our small CD patient cohort, including in active disease (Fig. S6*E*), *versus* what we saw in healthy samples (Fig. 3). We also noted varying levels of WGA intensity in CD HF-MUC2 with the least amount found in a CD remission sample (Fig. S6*E*). These studies suggest fecal MUC2 lectin profiling can be used to non-invasively detect potential glycosylation differences at the histological level. Collectively, these results indicate that sectioning of human fecal material can be used to non-invasively probe mucus structure, quality, and glycosylation *in situ* in relation to the microbiota in health and disease.

### Fecal MUC2 can be used to investigate structure-function relationships in mucin from IBD patients

**Figure 6:**
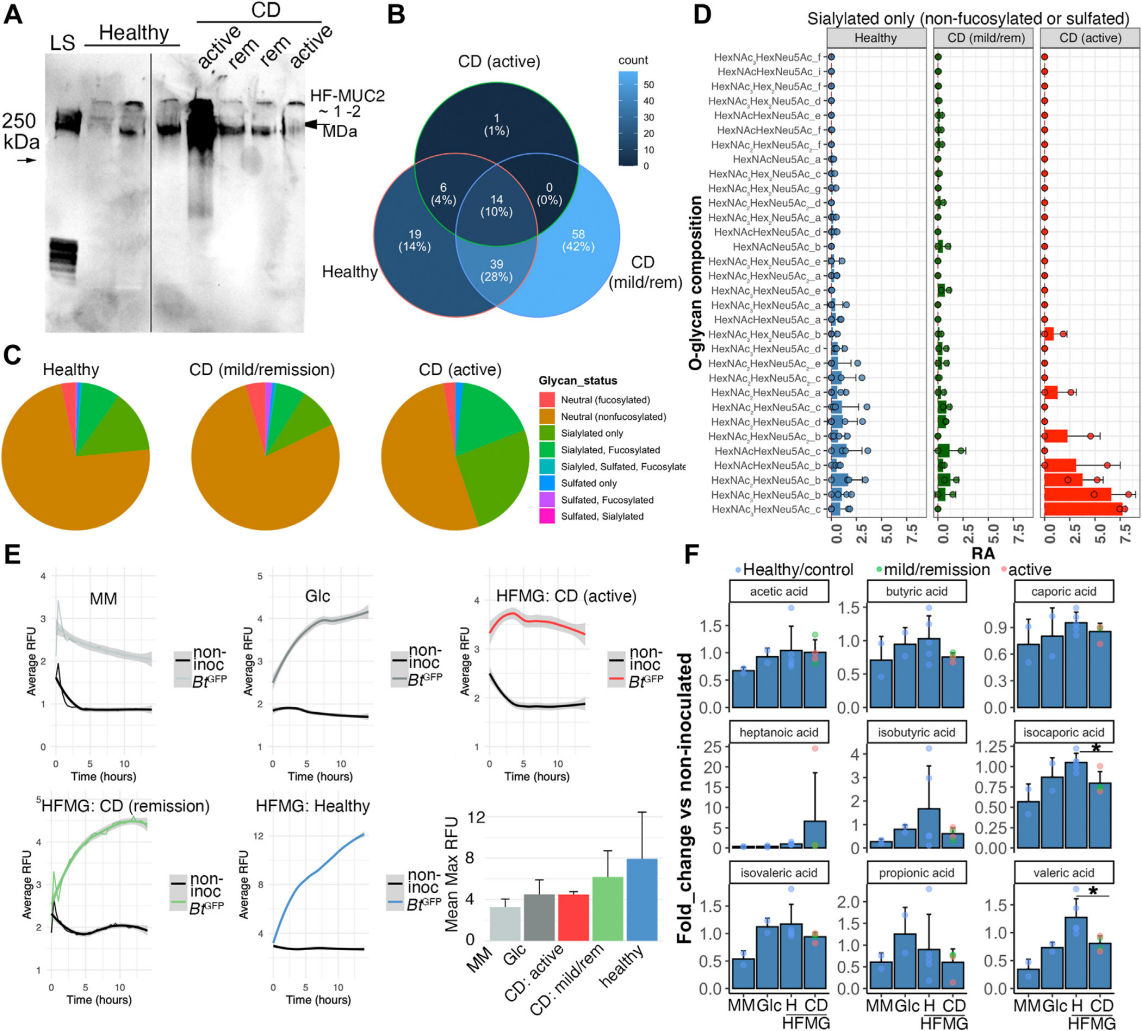
**Functional glycomic analysis of healthy *versus* Crohn’s disease-associated human fecal MUC2**. *A*, Western blot of human fecal (HF)MUC2 purified from feces of healthy *versus* CD patients, and separated by SDS-PAGE using TGX Any kD gels. Representative of two blots. *B*, Venn diagram showing overlap of common glycan structures from indicated sources. Unique and common glycans are indicated as total numbers with percent of total analyzed in parentheses. *C*, pie chart showing relative abundances of specific classes of glycans (indicated by color). CD = Crohn’s Disease. *D*, Barplot showing mean ± SD of individual sialylated glycans derived from extracted and purified fecal MUC2, each point a biologic replicate. *E*, growth curves of *Bacteroides thetaiotaomicron*^*GFP*^ (*Bt*^*GFP*^) in the presence of glycans from healthy *versus* CD patients. Barplot on bottom right is mean Max RFU ± SD (n= 2–4/group) to show variation in growth among biological replicates. HFMG = human fecal MUC2 O-glycans. *F*, Bar plot of mean ( ± SD) fold-change of short-chain fatty acid levels taken from spent supernatants of *Bt*^GFP^ grown in minimal media with or without human MUC2 O-glycans or Glucose (Glc) controls. Points indicate biological replicates, color-coded by disease status. CD, Crohn’s Disease; Glc, Glucose supplemented MM; H, Healthy; MM, minimal media. ∗*p*< 0.05, students *t* test, or Wilcoxon-rank sum test.

To determine if fecal MUC2 can be used to probe mucin structure-function relationships in healthy *versus* IBD samples, we extracted MUC2 from healthy (n = 5) and CD (n = 4) fecal samples for downstream electrophoresis and functional glycomic studies. SDS-PAGE showed the presence of a high molecular weight MUC2^+^ band in healthy and CD (active and remission) HF-MUC2 extracts that did not show any major differences in migration compared to healthy MUC2, although it was markedly more abundant in an active CD-derived sample (Fig. 6*A*). Notably, consistent with the *in situ* analysis, we did not detect MUC4 in the extracted HF-MUC2 prep *via* Western blotting (Fig. S6*D*), complementing our in-situ findings (Fig. S6*C*).

We next performed a deep analysis of MUC2 glycosylation in healthy *versus* CD HF-MUC2 *via* LC/MS. Glycans were released from extracted MUC2 *via* reductive β-elimination, and analyzed by negative ion mode electrospray ionization QToF-MS after their resolution by HPLC on a porous graphitized carbon column as previously described for other, comparable O-glycomic analyses (68). The results showed a diverse glycome could be found in HF-MUC2 preps from both healthy and CD samples, with healthy samples showing greater overlap with remission CD (28% total glycans) compared with active CD (4% total glycans) (Fig. 6*B*). These glycan differences overall were most starkly observed in HF-MUC2 O-glycans from the active CD patient samples, which showed greater overall relative abundance of glycan chains modified by sialic acid alone or with both sialic acid and fucose (Fig. 6*C*). To gain insight into where these differences lie, we compared the mean relative abundances of individual sialylated glycans between healthy, mild/remission CD, and active CD HF MUC2 (Figs. 6*D* and S7, *A*–*F*), finding that healthy and mild/remission CD samples largely mirrored each other, while active CD samples showed increases in specific isomers of tri-, tetra-, and pentasaccharides containing a Hex, Neu5Ac, and one or more HexNAcs (Fig. 6*D*). Similar active CD-specific increases in fucosylated + sialylated glycans were observed (Fig. S7*A*). These specific active CD samples also had lost much of their sulfation relative to the healthy and mild CD samples (Fig. S7, *C* and *D*). These studies suggest HF-MUC2 O-glycans can be used to discriminate between healthy and disease states, in line with recent studies (69).

### Fecal MUC2 O-glycans can link mucus barrier function and glycosylation status to microbial metabolism

We next sought to gain insight into how O-glycosylation of HF-MUC2 may be utilized to explore the functional impact of mucin alterations in health and disease. O-glycans are important for modulating microbial physiology including their growth and inflammatory potential (25, 37, 70), the latter in part *via* short-chain fatty acid (SCFA) production (69). Therefore, we designed an *in vitro* microtitre microbial assay to test how the differential glycan pools from healthy and CD HF-MUC2 impacted microbial growth and metabolism. Using HF-MUC2-derived O-glycans from healthy *versus* CD HF-MUC2 (remission and active disease), as the sole carbon source for fluorescence reporter *Bacteroides thetaiotaomicron-*GFP (*B*. *theta-*GFP) (71), we found that O-glycans on HF-MUC2 were able to support the growth of *B. theta-*GFP *in vitro* in minimal media comparable to glucose alone (Fig. 6*E*), confirming the O-linked oligosaccharides from human fecal MUC2 are functional. Notably, we saw growth in healthy O-glycans was fairly consistent, whereas it was less so within CD O-glycans, with the least growth in samples with active disease (Fig. 6*E*). To determine whether microbial growth resulted in differential production of SCFA (72), we harvested cleared supernatant 16 h after growth and subjugated it to gas chromatography/mass spectrometry (GC-MS) for a panel of SCFA. Compared to non-inoculated media of either treatment group, we found supernatants from *B. theta-*GFP cultured in the presence of HF-MUC2 O-glycans led to the production of several SCFAs including acetate, propionate, valerate, and to a lesser extent butyrate, although to varying levels, with acetate dominating. Because the scales were vastly different between the different SCFAs, we calculated the fold changes of SCFA in *B. theta*-GFP-inoculated *versus* non-inoculated media of each experimental treatment. We then compared the mean fold-change of each SCFA within our panel between healthy and CD HF-MUC2 samples. We found that healthy HF-MUC2 O-glycans were able to show significant increases in the production of valeric acid and isocaproic acid (*p* < 0.05, Students *t* test, n = four-fifths per group), and a trend toward increases in butyric acid *versus* CD HF-MUC2 independent of disease status (Fig. 6*F*). In contrast, CD HF-MUC2 O-glycans in these assays led to increased production of heptanoic acid, although not significant, and likely driven by the disease status (Fig. 6*F*).

Finally, to determine if fecal MUC2 O-glycans can be used to detect differences in Ulcerative Colitis (UC) samples, we obtained feces from control and UC patients (n = 3) and extracted MUC2. These samples were homogenized before analysis so imaging could not be done. However, intact HF-MUC2 could be visualized by SDS-UAgPAGE (Fig. S8*A*), and therefore amenable to biochemical characterization. SDS-UAgPAGE showed the presence of a high molecular weight MUC2^+^ band in the UC and healthy extracts (Fig. S8*A*). Glycans were limiting in these samples, so we chose to examine overall glycoprofiles *via* glycan fingerprinting by capillary electrophoresis (CE) which revealed the presence of peaks that were present in both UC samples but missing from the control (Fig. S8*B*). These studies are in line with recent studies that have shown differences in fecal mucin O-glycans in IBD patients (69). To functionally probe the UC HF-MUC2, we performed microbial growth assays of the purified HF-MUC2 O-glycans from healthy *versus* UC patients. As with the healthy and CD HF-MUC2-derived glycans (Fig. 6), we found that *B. theta*-GFP grew in all media that was supplemented with carbohydrates; however, unlike glucose and healthy HF-MUC2 O-glycans, growth in UC MUC2 O-glycans began to decline after 6 h but ultimately stabilized (Fig. S8*C*). Correspondingly, SCFA production from the UC HF-MUC2 O-glycans showed a differential profile of SCFA from *B. theta versus* healthy HF-MUC2 O-glycans and glucose alone. These changes included reduced acetic and propionic acids and increased butyric acid *versus* healthy controls (Fig. S8*D*).

Collectively, these studies indicate that MUC2 can be extracted from IBD (CD and/or UC) feces and structurally and functionally compared to understand how mucus thickness, penetrability, biochemistry, and function are altered in individual persons and potential populations.

## Discussion

Our work for the first time provides a platform to access abundant primary human MUC2 on human feces for visual, structural, and functional characterization. Using our platform, we found that mucus adherent to the feces can be readily i) visualized to probe microbe-mucus interactions and mucus structure *in situ*; ii) extracted for structural profiling and comparative O-glycomics and independent comparison with lectin profiling, and iii) functionally assayed to determine how glycosylation status influences host-microbe interactions in health and disease. Because this MUC2 is accessed in a completely non-invasive way (*i.e.* from excreted feces) and is perpetually renewed, this study opens up new opportunities for unprecedented access to this essential component of the mucosal ecosystem for further study.

Past studies have also explored mucus in feces, although to a limited extent. Fecal mucus has been observed by histochemical staining on fecal sections in the context of irritable bowel syndrome (43). Yamada *et al* (2019) looked at aspects of glycosylation and metabolism of fecal mucus preparations *ex vivo* (73). However, only recently have studies capitalized on the fecal adherent encapsulation model to specifically target MUC2. Robbe-Masselot *et al* (2023) for example have exploited fecal mucus by extracting it from healthy and IBD patients to determine whether mucin oligosaccharides can serve as novel biomarkers for disease, highlighting the potential of this platform (69, 73). However, the limitations of these analytical studies are that the quality and purification of the MUC2 were not ascertained for example by proteomics or Western blot; thus we are not certain MUC2 was analyzed (69, 73). In our studies, using standard mucus extraction (44) and tissue processing protocols, we are the first to show that we can now observe MUC2 visually, verify and analyze it biochemically, as well as assess its functionality in health and disease. Further, with our methods we can link structure to function potentially from the same individual, thus enabling the deepest characterization to date. To this end, we are the first to our knowledge to directly correlate primary MUC2 biochemical properties, glycosylation, and ability to induce commensal growth and characterize its metabolism in healthy and IBD patients. Because our study in essence is a proof-of-principle, our sample size was low; however, we are now increasing our sample sizes to understand the natural variation of the mucinome in the wider population and in healthy and diseased states.

Other methods to access primary human MUC2 for study include the use of explant studies of human biopsies or cell lines. For explant studies, a major advantage of this approach is that a truly native primary MUC2 can be functionally probed *via* confocal microscopy (74, 75). Indeed, these approaches, used both in humans and mice, have illuminated key functional defects in MUC2 in patients at risk of UC (*i.e.* before inflammatory symptoms), supporting the notion that a primary defect in mucus precedes disease development (28). However, these approaches are still limited in the need for biopsies which is a major barrier for many labs, as well as the limited sample area at which these explants are taken relative to the entire tissue. In regards to cell lines, to date, there are several transformed colonic cell lines that can produce gel-forming mucins including Methotrexate-adapted HT-29 cells (76, 77), and LS174T cells (78), the latter which can produce complex glycosylated MUC2 to explore host-pathogen interactions and glycomic changes in cancer (79). However, these cells are not produced by a healthy colon; and given recent studies highlighting significant goblet cell heterogeneity in mice and human colons (74), this cannot be relied upon as a normal and representative MUC2. Recently, adaptation of human colon organoids to air-liquid-interface systems can promote robust MUC2 production (80, 81, 82); however, it is unclear how similar ALI *versus* fecal MUC2 are, and the technical and economic challenges associated with organoid culture present an important barrier for many labs. The use of fecal material enables cost-effective and efficient isolation of primary MUC2 from individuals and provides an easier opportunity to explore MUC2 biology across a wider spectrum of society, stratified by virtually any variable (health status, sex, age, ethnicity) according to the research question.

We used our fecal MUC2 analytical platform for cross-species comparisons, one of its many applications. Our goal with pig colonic MUC2 comparison was to assess sulfation status, which is a regulator of glycan breakdown by mucus-foraging species (34); Pig colonic mucin is rich in these modifications (34); therefore we analyzed sulfation of HF-MUC2 to gain insight into the prevalence of this modification in a human. Our porcine colon MUC2 was enriched with sulfated glycans *versus* HF-MUC2, at least in our direct comparison. This suggests human MUC2 may be more susceptible to glycosidase activities in the presence of key glycan-foraging symbionts such as *B. theta* (34). Our platform can allow for such hypotheses to be routinely tested. With mice, we compared mucus structure *in situ*. Although the human and mouse fecal-adherent MUC2 showed similar barrier function *in situ*, we noted several differences based on lectin staining patterns. First, there was no obvious b1/b2 system in human fecal mucus as seen in mice. In mice, the MALII (α2-3 Sia or sulfated Gal) binds the distal colon b2 layer, while the major b1 layer is MALII-negative. We found in humans the major barrier layer is strongly bound to MALII. This does not rule out the existence of the b1/b2 system as humans and mice are known to have different glycoprofiles, most notably the dominance of core three or 4-based O-glycans in humans *versus* core one- and 2-based in mice (61), so the b1/b2 system in human may require a unique combination of lectins. Alternatively, the b2 layer in humans may be more adherent to tissues, which remains to be determined. However, the fact we can visualize a clear and continuous barrier layer in humans demonstrates at the very least an adherent mobile mucus barrier phenotype is conserved between mice and humans (and likely most other species).

A second notable difference between rodents and humans was regarding lectin profiles. While mouse fecal mucus strongly stains for UEA1 (Fucα1-2Gal) and SNA (SNAα2-6Gal), these were notably absent in most healthy human fecal mucus analyzed. The weak/absent UEA1 result is consistent with previous studies showing weak UEA1 staining in human colon goblet cells (60), and therefore it is unlikely a result of non-Secretor (FUT2-null) status. The antigen may be masked due to sialylation or fucosylation at the α1,3 position. Desialylation did not unmask the UEA1 epitope, but we cannot rule out α1-3-linked fucose as a blocker. Notably, the samples were robustly fucosylated as demonstrated by AAL staining, which recognizes α1,three-fourths linked glycans (57). Regarding SNA staining, this lectin binds terminal Siaα2-6Galβ1-3/4GlcNAc residues (Type 1 & 2 LacNAc) (83), although whether this preferentially binds this epitope on N-glycans over O-glycans is not definitively settled (58, 83). However, the results are consistent with previous reports showing an absence of SNA binding in normal colon epithelia, even after mild saponification to remove O-acetyl groups that block SNA (84). This suggests the SNA epitope, readily seen in mouse Muc2, may be less readily found in healthy human fecal MUC2.

A key implication of our analysis is that primary human MUC2 and its interaction with the microbiota clearly represent the most accurate interaction if we want to understand how the mucus system in humans impacts the function of key symbionts. To this end, we assayed the ability of human MUC2 oligosaccharides to promote growth and SCFA metabolite production from the well-characterized mucin utilizer *B. thetaiotaomicron* (34, 85), finding MUC2 O-glycan-dependent growth indeed produced an SCFA profile different from glucose alone, in line with Hino *et al* (2020) with porcine gastric mucin O-glycans (72). Interestingly, we provide direct evidence linking altered glycosylation of CD and UC fecal MUC2 to differential growth and SCFA profiles, in line with Yamada *et al* (2019) (73). This shows we can compare the functionality of MUC2 from different states of health to gain insight into the relationship of mucus to dysbiosis of the microbiota.

Among the most important things we learned in this study is the variation we observed in the mucus barrier thickness from independent samples. Using our thickness-along-a-distance metric, the thickness of the fecal mucus layer varied around the periphery substantially in some samples and little in others (Fig. 1). The basis for this is unclear, as is its biological significance. For example, it may reflect points of barrier disruption, or it may be an artifact of sample collection, movements within the colon, or processing. There was also substantial variation in mean thickness between individuals both in humans and mice. This suggests careful thought of power analysis must be incorporated into the experimental design to ensure appropriate statistical rigour for formal clinical and basic research questions.

Our initial analysis of healthy *versus* IBD HF-MUC2 glycomes indicates the potential for disease screening and characterization. For example, there was remarkable consistency of glycoprofiles between independent HF-MUC2 samples from healthy and mild/remission CD (6 in total). This is in agreement with the findings by Larsson *et al* (2011) showing a complex but similar overall glycoprofile of MUC2 extracted from biopsies across 25 healthy individuals (52). Based on this consistency, the clinical question to see if HF-MUC2 glycans can be used to detect differences in healthy *versus* CD we believe also accurately captured a major shift in the glycome of active CD samples, which both showed a similar increase in sialylated and/or sialylated+fucosylated glycans, and the fact that sialylation-specific SNA seemed to ready label an active CD sample. This is in line with studies showing inflammation can upregulate sialyltransferases in colon cells (86). However, we will clearly need to increase our sample sizes to see how significant these differences are in the wider population and to compare with the literature (69).

There are some potential limitations of using fecal MUC2 for downstream analysis, including the impact of stool consistency, collection protocol, and unknown status of mucin metabolism at the time of collection. First, regarding consistency, fecal material can be solid or loose depending on hydration levels, in both healthy and diseased individuals (43). We found that undisturbed solid fecal masses showed the most continuous mucus layer along the periphery of the fecal mass, whereas this was lacking in loose material. This suggests that the mucus layer may not always be functional even in healthy states, and may point to the barrier-independent functions of MUC2 (*e.g.*, preventing dysbiosis, promoting anti-inflammatory metabolites) (22). Importantly, in loose material, clear strands of mucus could be observed, consistent with a previous study (43). However, this mucus is extractable and its properties were similar to MUC2 extracts from solid feces as shown by Western blotting. This indicates that while imaging of mucus barrier functions may be challenging in loose stools, MUC2 can still be readily analyzed *via* other methods. Second, collection protocols (*e.g.*, storage conditions) may influence mucus quality. Typically, immediate fixation in Carnoy’s fixative or storage at −80 °C would represent best practices. However, these present potential logistical barriers when working with human volunteers. We found simply preserving feces at −20 °C prior to analysis and then collecting samples for histology readily preserves the mucus barrier. This property allows for flexibility of timing when acquiring fecal samples from volunteers. It also potentially allows the inclusion of this analysis in established fecal analysis workflows, for example, fecal calprotectin assays in IBD, microbiota analysis, fecal occult tests, and others (87, 88).

Finally, it is possible that the overall glycome observed may not reflect a completely unmetabolized glycome due to microbial degradation, or may reflect glycans from potential O-glycosylated co-purified glycoproteins. Regarding the potential of microbial metabolism, the evidence suggests the glycome is likely overall intact given the size of the purified mucin bands (1 – MDa range as expected for mature mucus) (45) and the lack of clear degradation products. This is consistent with our extraction procedure specifically targeting the polymerized mucus barrier that microbes do not penetrate. In support of this, the glycome of fecal Muc2 of mice contained large numbers of intact glycans that were similar to those observed in the tissues (37). With respect to the co-purified proteins; the main ones observed, including SMR3B (Accession: AAH94707.1), and CELB (Accession:KAI4079020.1) lack the Ser/Thr-rich densely O-glycosylated mucin domains found on MUC2, and thus would only contribute to a minor fraction of O-linked oligosaccharides compared to MUC2 if they harboured any O-glycosites at all. However, it is notable that SMR3B is evolutionarily related to MUC2 (50). Collectively, this supports the fecal MUC2 glycome of the mucus barrier layer as representative the mature intact glycome secreted by goblet cells into the lumen.

In sum, our studies outline a comprehensive non-invasive MUC2 analytical platform. We have only begun to explore the potential to understand the complex biochemistry and biology of mucins like MUC2. We are confident this can be used to provide novel insights into MUC2 structure and function in cross-sectional and longitudinal studies in health and disease and in response to virtually any intervention.

## Experimental procedures

### Animals

*Mouse*: 6 to 8 week-old C57BL/6 mice male mice (n = 4) were purchased from The Jackson Laboratory (#000664). Animals were fed standard chow (PicoLab Rodent Diet 20; LabDiet 5053). Animals were housed in specific pathogen-free facilities (SPF). All animal experiments were approved by the Institutional Animal Care and Use Committee of the University of British Columbia (Protocol #A10–0073). *Pig:* No live pigs were used in this study; the porcine colon mucus was obtained from a local British Columbia abattoir where staff removed the tissue from a mature pig, gently rinsed it with tap water, and immediately transferred the whole colon to ice. Only one pig was used for this study. The colon was subsequently stored frozen at −20 °C prior to mucus extraction (below).

### Fecal collection

Fresh fecal pellets were obtained from mice either by collection from the surface of a clean cage immediately after defecation, or through direct collection into a sterile microtube through standard scruffing techniques approved by the UBC Animal Care Committee. Fecal material from human volunteers was obtained with prior ethical approval by the UBC Clinical Research Ethics Board (Protocols: H21–3534, H16–03300, and H22–02645). The human studies were performed in accordance to the Declaration of Helsinki. Healthy donors were adult males or females ages 22 to 44 (n = 5 in total), either Caucasian, south Asian, or Middle Eastern (H21–03534). For the CD patients (ethics: H22–02645), fecal samples were from biological male and female patients between 40 to 72 years of age with moderate (active) disease or in remission/mild disease and not on antibiotics. The diseases ranged from 15 to 469 months duration. Ulcerative colitis (UC) fecal samples were from patients with active disease (n = 5 in total, male and female, ages 39–56, not on antibiotics: ethics approval H16–03300), and were collected and homogenized first and ∼2 g were used for mucus extraction using healthy feces prepared in the same way as technical control. Due to limiting glycans per sample for UC, the glycans were pooled for downstream microbial assays. In each case, samples were collected in a plastic bucket and either immediately processed for fixation and imaging (see immediately below) and mucus extraction (see “Mucus/MUC2 isolation” below) or stored at −20 °C or −80 °C.

### Processing for histology

For fixation, either small boli were taken as a whole, or small fecal sections were carefully collected from the fecal surface using a single-edge disposable blade, and placed in Carnoy’s solution (60% methanol; 30% chloroform; 10% glacial acetic acid) for minimum 2 h at 4 °C, maximum 24 h, and processed overnight for paraffin embedding. Samples were oriented to get cross-sections along the fecal surface. 5 μm Carnoy’s fixed paraffin embedded (CFPE) sections were collected. The procedure was identical for loose fecal material. For fecal samples in which prior freezing was tested, we first took surface slices from fresh material, immediately froze them for 2 weeks at −20 °C, and then placed the frozen slice directly in Carnoy’s fixative for processing as above.

### Combined immunostaining and fluorescence *in situ* hybridization (FISH)

Dual MUC2-FISH staining is a two-step process performed as previously described (37). First, deparaffinized sections were incubated at 37 °C overnight with a Texas Red–conjugated universal bacterial probe EUB338 (5′-GCTGCCTCCCGTAGGAGT-3′; bp 337–354 within bacteria EU622773; Eurofins MWG Operon) in hybridization buffer (20 mM Tris-HCl, pH 7.4, 0.9 M NaCl, 0.1% sodium dodecyl sulfate). The sections were rinsed with wash buffer (20 mM Tris-HCl, pH 7.4, 0.9 M NaCl) and PBST (1 × PBS pH 7.0 + 0.05% Tween-20) twice each. Second, sections were then incubated with rabbit polyclonal anti-MUC2 (41) for 2 h at room temperature, or overnight at 4 °C, rinsed with PBST, and then incubated with Alexa Fluor (AF)488 or DyLight 647- labeled donkey anti-rabbit IgG (8 μg/ml, Jackson Immunoresearch). For other imaging (Fig. 5), we used an AF488 pre-conjugated commercial mouse monoclonal IgG_1_ κ antibody against human MUC2 C-terminal amino acids 4880 to 5179 (Mucin 2/MUC2 Antibody (F-2); sc-515032 AF488) for direct immunolabeling *in situ*. Slides were washed three times in dH_2_O, sometimes stained with 20 ng/ul DAPI for 5 min, washed again in dH_2_O, and mounted with Fluoroshield (Sigma, SKU F6182–20 Ml). The sections were imaged by widefield or confocal microscopy.

### Lectin and immunostaining

Deparaffinized and rehydrated CFPE sections of colons or fecal material were incubated with streptavidin and biotin-blocking solution (Vector Laboratories) to block endogenous biotin and avidin, followed by incubating in CAS-Block (Invitrogen) serum-free blocking buffer for 10 min at 4 °C. Sections were then incubated with a combination of lectins or lectins with MUC2 as described in our results. The lectins used were biotinylated *A. aurantia* lectin (AAL, five ug/ml in ADB, B-1395–1, Vector Laboratories), Wheat-Germ Agglutinin-Fluorescein (WGA-Fluorescein 5 ug/ml in ADB, FL-1021, Vector Laboratories), biotin or FITC-conjugated Tomato lectin (5 ug/ml in ADB, B-1175–1, Vector Laboratories); Biotinylated or Rhodamine/DyLight 350/DL-647-conjugated *U. europaeus agglutinin* I (UEA1, 4 ug/ml, RL-1062–2 and others, Vector Laboratories), and biotinylated or FITC-*S. nigra agglutinin* 1 (SNA-1, 10 ug/ml, FL-1301–2, Vector laboratories), biotinylated *M. amurensis* lectin II (MALII, 3 ug/ml, B-1265–1, Vector Laboratories), and/or polyclonal anti-MUC2 (41). For some studies, we also used mouse monoclonal anti-human MUC5AC IgG (45M1) (Santa Cruz #sc-21701), and mouse monoclonal anti-MUC4 IgG (1G8) (Santa Cruz #sc33654) to co-stain with MUC2-488 above. Slides were incubated for 2h at room temperature, or overnight at 4°C, rinsed with PBS-T (1× PBS + 0.05% Tween-20), and then incubated with appropriate secondary detection agent using Alexa Fluor 350/488/594/or 647-conjugated Streptavidin (5 μg/ml, Jackson Immunoresearch) or Alexa Fluor 594 or DyLight 647- labeled donkey anti-rabbit IgG (5 μg/ml, Jackson Immunoresearch) for 1 h at room temperature in the dark. Slides were washed three times in dH_2_O, stained with 20 ng/μl DAPI for 5 min, washed again in dH_2_O, and mounted with Fluoroshield (Sigma, SKU F6182–20Ml). In all cases where directly conjugated lectins were co-stained with a reagent requiring secondary detection, the directly conjugated lectin was incubated with the secondary detection agent (*e.g.*, Streptavidin). In all cases where secondary detection is required for both reagents (*e.g.*, Muc2-MALII staining), the primary and secondary agents were paired. The slides were used for subsequent confocal imaging and tiling (described below).

### Mucus/MUC2 isolation

Mucus isolation from human feces, mouse fecal pellets and pig colon was performed as previously described with some modifications (37). Briefly, the frozen pig colon was partially thawed and opened up longitudinally and luminal content was removed carefully. The secreted mucus was gently scraped off with a glass slide. Secretions were dispersed using 10 ml guanidium chloride (GuCl) extraction buffer (6 M GuCl, 0.1 M Tris pH 8.0, 1 mM EDTA) containing 2× cOmplete Protease Inhibitors (Millipore-Sigma). For mucus isolation from fecal material, 5 to 10 g of fresh or frozen feces were placed directly into a 50 ml tube containing 10 ml guanidium chloride extraction buffer with 10× cOmplete Protease Inhibitors. The solution was vortexed. All samples were then extracted overnight at 4 °C on a rotator. The samples were pelleted by centrifugation and pellets were resuspended and re-extracted with an equal amount of guanidium chloride extraction buffer. Following re-extraction and removal of the supernatant, samples were reduced twice with 100 mM dithiothreitol (DTT) suspended in guanidium chloride (GuCl) extraction buffer to solubilize the mucin (once overnight at 37 °C, the other for 4 h at 37 °C). Soluble mucins were then alkylated with 250 mM iodoacetamide on a rotator overnight at room temperature in the dark. The reduced and alkylated mucins were dialyzed into ddH_2_O, using Repligen 100 kDa MWCO Dialysis Device or tubing. Following dialysis, a subsection of samples was treated with Pierce High Capacity Endotoxin Removal Resin in batches (1 ml of resin to 1–10 ml of mucins) packed into 10 ml Pierce Centrifuge Columns. The dialyzed material was then freeze-dried overnight and stored at −20 °C until being analyzed *via* electrophoretic or glycomic approaches described below.

### Gel electrophoresis

Analysis of mucins was performed using composite sodium dodecyl sulfate UreaAgPAGE (SDS-UAgPAGE) (89) or precast Mini-PROTEAN Tris-Glycine Extended (TGX) Any kD Gel (BioRad) as indicated in figure legends. The composite SDS-UAgPAGE gel was made up of a gradient of 0.5 to 1.0% agarose, 0 to 10% glycerol, and 0 to 6% acrylamide. The running buffer consisted of buffered boric acid (192 mM boric acid, 1 mM EDTA, 0.1% SDS, pH 7.6). Following electrophoreses for 4 h at 110 V on ice, the gels were stained by PAS using the Pierce Glycoprotein Staining Kit (ThermoFisher Scientific; according to manufacturer’s instruction) or processed for Western blotting. For Western blotting, mucins were transferred onto a polyvinylidene difluoride (PVDF) membrane *via* wet transfer at 100 V for 1 h on ice using standard Tris-Glycine transfer buffer with 20% methanol. The PVDF membranes were blocked by 5% skim milk in TBST buffer for 1 h and then incubated with 1:2000 rabbit anti-MUC2 antibody (anti-LS174T) diluted in the blocking solution. We also probed select membranes for mouse anti-MUC4 (details above) at 5 ug/ml. For lectin blots, PVDF membranes were first blocked with a Streptavidin-Biotin blocking kit (Vector Laboratories; according to the manufacturer’s instructions) then incubated with either biotinylated SNA (2 ug/ml), succinylated WGA (2 μg/ml), UEA1 (2 μg/ml), AAL (2 μg/ml) or LEL (2 μg/ml) in TBST at 4 °C overnight. Membranes were washed with TBST, incubated with either 1:2000 HRP-conjugated goat anti-rabbit IgG (for MUC2) or 1:2000 HRP-conjugated streptavidin (for lectins) for 1 h at room temperature, then detected with Pierce SuperSignal Enhanced Chemiluminescence reagent (Thermo Scientific) and developed using a ChemiDoc MP system (BioRad).

### Saponification and sialidase treatments *in situ*

Deparaffinized and rehydrated CFPE sections were sequentially saponified using 0.1 M sodium hydroxide (NaOH, 15 min) or 0.5% potassium hydroxide (KOH) in 70% EtOH for 30 min, which removed O-acetyl groups that can mask lectin and neuraminidase activities. For some assays, no saponification was done. We then used either α2-3 neuraminidase S from *Streptococcus pneumoniae* (#P0743, New England Biolabs; 100U/section in 1× Glycobuffer), or an α2-3,6,8 neuraminidase from *Clostridium perfringens* (#P0720, New England Biolabs) to target overall sialylation. Both enzymes were incubated on tissues overnight at 37 °C in a humidified chamber. Negative controls were samples treated with reaction buffer only. Negative control or neuraminidase-treated samples were then stained with MALII, SNA, and WGA along with UEA1 as a control for glycosidase specificity, and analyzed by epifluorescent microscopy.

### Widefield and confocal imaging

To tile-image the fecal sections, an Olympus IX81 microscope equipped with a motorized stage to enable tiling at 4× and 10× magnifications was used. Tiled images were stitched together using Olympus cellSens Dimension software (Version 3.1). A Leica DMi8 confocal microscope was used for confocal imaging where Z-stacked confocal images were acquired with a 20× dry objective, or a 60× oil-immersion objective, with optical sections taken at 0.3 to 0.5 μm intervals. The images were analyzed using Imaris 9.9.1 (Bitplane). For epiflourescent images, an EVOS (ThermoScientific) microscope was used.

### Quantitative imaging

#### *In situ* mucus thickness measurements along a distance

The input image is a tiled cross-section of a CFPE fecal pellet stained with anti-MUC2 (arrows; see also Fig. 1*B*). This image is then processed in Fiji/ImageJ (90) through straightening, splitting channels, and quantitation of x and y values along the length which may include the entire circumference if section quality is good. If processing artifacts occur along the length, ROIs are excluded. In the Fiji/ImageJ plot profile function, the x values represent distance in pixels across the image. The y values represent the mean intensity of the pixels. Therefore, to convert the mean intensities to absolute numbers of pixels, a binary mask was created on an 8 bit image, generating completely black or white pixels (with intensity values of 0 or 255, the max intensity of an 8 bit image). The proportion of positive pixels in the y direction was then calculated by dividing the mean intensity by the max 255. The proportion of positive pixels was then multiplied by the height of the image in pixels, giving the absolute number of positive pixels in the y direction. This data was then converted to a thickness value by multiplying by the known scale (microns/pixel) for that image. This enabled direct calculation of thickness *versus* distance in microns. To perform these calculations, the raw data from ImageJ plot profile is copied into an Microsoft Excel file and processed in RStudio (91) through an in-house R script that imports the data (XLConnect), and converts pixels to microns (dplyr) based on the scale recorded in Fiji and the conversion factors above, and then plotted using ggplot2 package (92). Values were verified by manual measurements in ImageJ on subsamples of the mucus layer and comparing with the script.

### Bacterial distance from mucosal surface or bottom of mucus

For confocal images stained with FISH probes and mucus markers (Lectins or MUC2 antibody, described above), the distance of each bacterium (∼300–2000/image) to the mucosal surface (or bottom of mucus for fecal mucus studies) was determined using the Surfaces and Spots functions within Imaris software (Bitplane; V9.9.1). Briefly, Imaris Spots accurately (∼90–95% precision) pinpointed each bacterial cell and assigned them a unique ID. The Surface function was used to generate a new surface (called “Tissue”) for the mucosal tissue, either automatically, or *via* careful manual drawing of contours along a clearly visible mucosal surface. The software then calculated the distance of each bacterium to the mucosal surface using the “distance to nearest surface = Tissue” calculation. The calculations were verified using a subset of manually-measured bacteria. Data was exported into Microsoft Excel and then to RStudio for data visualization using ggplot2 (92) to generate a density plot of the frequency distribution of the absolute numbers of bacteria at a given distance to the bottom of the mucus layer.

### 384-Well Fluorescent bacterial growth assay

GFP-expressing *B. thetaiotaomicron* (*Bt-*GFP) (71) were grown under anaerobic atmospheric conditions (10% CO_2_, 5% H_2_, 85% N_2_) at 37 °C overnight in BHI broth supplemented with L-cysteine (1 g/L), Hemin-Histidine solution (0.225 M Histidine, 2.2 mM Hemin; 1 ml/L), Resazurin solution (0.1%; 1 ml/L), and NaHCO_3_ (0.2%). 2× Minimal Media (modified from (93)) consisted of KH_2_PO_4_ (200 mM), NaCl (30 mM), (NH_4_)_2_SO_4_ (17 mM), L-cysteine (16 mM), MgCl_2_•6H_2_O (0.2 mM), FeSO_4_•7H_2_O (0.0028 mM), CaCl_2_•2H_2_O (0.108 mM), Vitamin K1 (2 μg/ml), Vitamin B12 (10 ng/ml), Hemin-Histidine solution (0.225 M Histidine, 2.2 mM Hemin; 2 ml/L), and NaHCO_3_ (0.4%). 140 μl each of carbon-supplemented minimal media treatments were prepared under anaerobic conditions by diluting 70 μl 2× Minimal Media in sterile water and/or a specified carbon supplement. Glucose was added to a final concentration of 5 mg/ml, whereas all human glycans were added to a final concentration of 10 mg/ml. 140 μl of carbon-absent minimal media (MM) was also prepared *via* the addition of sterile deionized H_2_O. 500 μl of overnight *Bt*-GFP culture was collected and centrifuged at 3000RPM for 5 min to pellet bacteria. The supernatant was removed, and the pellet was gently re-suspended in 500 μl of 2× MM. 2 μl of the re-suspended culture was added to a 70 μl aliquot of each treatment condition. 20 μl of each non-inoculated and inoculated treatment was pipetted into a black 384-well flat-bottomed microtiter plate (Corning 384-well microplate, CLS3821BC) in replicates of three. The plate was then carefully sealed using a transparent gas impermeable adhesive plate seal (BioRad Microseal ‘B’ seal), and removed from anaerobic conditions. The plate was loaded into a Varioskan LUX Multimode Microplate Reader (Thermo Scientific) and fluorescence (ex. 488, em. 520) was read for ∼16 h at 20-min intervals at 37 °C. Relative fluorescence unit (RFU) measurements over time were exported and used to construct growth curves using R (Version 4.2.2).

### SCFA analysis

Spent cultures from the 384 well growth assay were pooled according to the treatment group, cleared by centrifugation, and then cleared supernatants (60 μl) were stored at −80°C until analysis. SCFAs (acetic, propionic, heptanoic, valeric, caproic, and butyric acid) were analyzed from culture supernatants by gas chromatography (GC) as described previously (94). In brief, 700 μl of −20 °C isopropyl alcohol containing 2-ethylbutyric acid at 0.01% *v*/*v* as internal standard was added to each 60 μl sample and then vortexed for 1 to 2 min. The samples were then incubated at room temperature for 15 min to facilitate SCFA diffusion into the solution. The sample was subsequently centrifuged at 11,000 rpm at 4C for 15 min, and the supernatant (∼600 μl) was transferred to gas chromatography (GC) autosampler vials and sealed. The autosampler vial content was injected into a Trace 1300 Gas Chromatograph, equipped with a Flame-ionization detector, with an AI1310 auto sampler (Thermo Fisher Scientific) in splitless mode. A fused silica Rtx-WAX (Restek) column 30 m × 0.32 mm i.d. coated with 0.5 μm film thickness was used. Helium was supplied as the carrier gas at a flow rate of 1.8 ml/min. The initial oven temperature was 100°C, maintained for 5 min, raised to 240 °C at 4 °C/min, then held for 15 min. The flame-ionization detector and the injection port temperature were 280 °C and 250 °C, respectively. The flow rates of hydrogen, air and nitrogen as makeup gas were 35, 350 and 30 ml/min, respectively. Peak areas and retention times were then calculated using Chromeleon seven software (Bannockburn) and compared against standards (Sigma-Aldrich).

### Proteomics

#### Protein Determination and manual In-Solution digestion

Proteomics was performed by University of Victoria proteomics services. The proteins in the samples were precipitated with six volumes ice cold acetone at −20 °C overnight. The samples were spun for 10 min at 4 °C at 17000*g* in a centrifuge and the acetone was removed by pipette. 50 μl (25 mM) ammonium bicarbonate/4 M urea) was added to the samples; sample H2 had a slightly larger pellet, so 100 μl (25 mM ammonium bicarbonate/4M urea) was added to that sample.

A Bradford assay was used to determine the protein quantitation in each sample. The disulfide bonds were reduced using dithiothreitol at a final concentration of 10 mM. Samples were incubated at 37 °C for 30 min prior to the addition of iodoacetamide in 25 mM ammonium bicarbonate to a final concentration of 40 mM. After incubation for 30 min in the dark at room temperature, dithiothreitol in 25 mM ammonium bicarbonate was added to a final concentration of 40 mM to quench the alkylation. 25 mM ammonium bicarbonate was added to reduce the urea concentration to 0.95 M. Trypsin (3 μg) in 25 mM ammonium bicarbonate was added to each sample and incubated overnight at 37 °C. The samples were acidified with formic acid and stored at −20 °C until ready for analysis. The peptides were de-salted using C18 Stagetips eluted with 80% Acetonitrile/0.1%Formic acid water, speed vacuum concentrated and rehydrated with 25 μl 2% ACN/98% water/0.1%FA prior to LC-MS/MS analysis.

#### LC-MS/MS analysis: Orbitrap Fusion

The peptide mixtures (5 μl of 25 μl) were separated by on-line reverse phase chromatography using a Thermo Scientific EASY-nLC 1000 system with an Acclaim PepMap100 C18 (100 μm I.D., 2 cm length, 5 μm, 100 Å) reversed-phase pre-column, and an AcclaimPepMap100 C-18 (75 μm I.D., 15 cm length, 3 μm, 100 Å, Thermo Fisher Scientific) reversed phase nano-analytical column at a flow rate of 300 nl/min. The chromatography system was coupled on-line with an Orbitrap Fusion Tribrid mass spectrometer (Thermo Fisher Scientific) equipped with a Nanospray Flex NG source (Thermo Fisher Scientific). Solvents were A: 2% acetonitrile, 0.1% formic acid and B: 90% acetonitrile, 0.1% formic acid. After a 348 bar (∼4 μl) pre-column equilibration and 348 bar (∼4 μl) nanocolumn equilibration, samples were separated by a 120-min gradient (0 min: 5%B; 100 min: 42%B; 15 min: 100%B; hold 5 min: 100%B). The Orbitrap Fusion instrument parameters (Fusion Tune 3.3 software) were as follows for Orbitrap (OT-MS) iontrap (IT- MS/MS) with HCD fragmentation: Nano-electrospray ion source with spray voltage 2.55 kV, capillary temperature 275 °C. The acquired survey MS1 scan range was 350 to 1800 *m*/*z* in profile mode, at a resolution of 120,000 (full width at half maximum at *m*/*z* 200); the siloxane *m*/*z* of 445.12002 was used as lock mass for internal calibration. Data-dependent acquisition Orbitrap survey spectra were scheduled at least every 3 s, with the software determining “Automatic” number of MS/MS acquisitions during this period. The automatic gain control (AGC) target value for FTMS was set to Standard (400,000 counts) and automatic maximum fill time to ensure optimal sensitivity and cycle time. The most intense ions charge state 2 to 5 exceeding 20,000 counts were selected for HCD fragmentation in the ion routing multipole. Monoisotopic Precursor Selection (MIPS) was enabled and Dynamic exclusion settings were: repeat count: two; repeat duration: 10 s; exclusion duration: 15 s with a 10 ppm mass window. The data-dependent (ddMS2) IT HCD scan used a quadrupole isolation window of 1.6 Da; Iontrap rapid scan rate, automode normal m/z range, centroid detection, one microscan, Auto maximum injection time, AGC target (Standard) 10,000 counts and stepped HCD collision energy of 28,30 and 32%.

#### Data analysis parameters

For database searching, tandem mass spectra were extracted and charge state deconvoluted by Proteome Discoverer version 2.5. Deisotoping was not performed. All MS/MS samples were analyzed using Sequest (Thermo Fisher Scientific; version IseNode in Proteome Discoverer 2.5.0.400). Sequest was set up to search contaminants.fasta; *Homo sapiens* (sp_canonical TaxID = 9606) (v2021–10–30) (2021–10–30, 20,559 entries) assuming the digestion enzyme trypsin. Sequest was searched with a fragment ion mass tolerance of 0.60 Da and a parent ion tolerance of 10.0 ppm. Carbamidomethyl of cysteine was specified in Sequest as a fixed modification. Oxidation of methionine and acetylation of the *N*-terminus were specified in Sequest as variable modifications.

For Criteria for protein identification, Scaffold (version Scaffold_5.1.0, Proteome Software Inc) was used to validate MS/MS-based peptide and protein identifications. Peptide identifications were accepted if they could be established at greater than 95.0% probability by the Percolator posterior error probability calculation (95). Protein identifications were accepted if they could be established at greater than 95.0% probability and contained at least two identified peptides. Protein probabilities were assigned by the Protein Prophet algorithm (96). Proteins that contained similar peptides and could not be differentiated based on MS/MS analysis alone were grouped to satisfy the principles of parsimony. Proteins sharing significant peptide evidence were grouped into clusters.

### Glycan analysis by high-performance liquid chromatography-mass spectrometry (HPLC-MS)

#### Glycan extraction

Human or murine fecal or porcine colon mucus samples, extracted and purified as described above, were lyophilized into 1.5 ml screw-capped centrifuge tubes (Sarstedt). Mucin O-glycans were released by reductive β-elimination in 100 μl 50 mM NaOH containing 1 M NaBH4 at 45 °C for 16 h. Samples were cooled to 25 °C and neutralized by adding 10 μl aliquots of 2 M acetic acid—vortex-mixing after each addition—until bubbling stopped. Samples were made up to 1 ml with ultrapure H_2_O and dried in vacuo on a Savant SPD121P SpeedVac concentrator connected to a Savant RVT5105 refrigerated vapor trap (ThermoFisher Scientific) until approximately 50 μl solution remained. Ultrapure H_2_O was added to 200 μl and samples were lyophilized overnight. O-glycans were recovered from the dried material by solid phase extraction (SPE). SPE was performed using 250 mg Supleco ENVI-Carb graphitic carbon cartridges (Sigma-Millipore). Cartridges were conditioned with 3 ml aqueous 80% acetonitrile (ACN) with 0.1% trifluoroacetic acid (TFA) followed by 6 ml ultrapure H_2_O; positive pressure at the top of the tube was used for all SPE procedures. Crude glycan samples were dissolved in 500 μl ultrapure H2O and loaded onto the cartridges, followed by 200 μl ultrapure H_2_O used to rinse the sample tube. The cartridges were washed with 3 ml ultrapure H_2_O, and reduced O-glycans were eluted using four sequential 550 μl aliquots of aqueous 50% ACN with 0.1% TFA. Eluates were dried in vacuo to approximately 50 μl, transferred to 200 μl polypropylene HPLC vial inserts, and lyophilized. Samples were re-dissolved in 30 μl ultrapure H_2_O prior to analysis.

HPLC-MS. HPLC was conducted on an Agilent 1290 Infinity system (Agilent Technologies) with a 1290 Infinity binary pump, a 1290 Infinity autosampler, and a 1290 Infinity column compartment. Analytes were separated on a Hypercarb 100 mm × 2.1 mm column (3 μm particle size) (ThermoScientific) at 60 °C. Samples were analyzed using an injection volume of 5 μl and a flow rate of 0.450 ml/min. Mobile phases A and B were H_2_O and ACN, respectively albeit data were collected using two different mobile phase additives previously used for glycomic analyses: 0.1% ammonium formate (62, 97) or 10 mM ammonium bicarbonate (68) (in both cases additives were added to both A and B). The following gradient elution was used for all separations: 0 to 15 min, 0 to 15% B; 15 to 22.5 min, 15 to 25% B; 22.5 to 25 min, 25 to 40% B; 25 to 25.2 min, 40 to 98% B. The column was then washed with 98% B for 2.6 min and equilibrated with mobile phase A for 3 min prior to the next injection. MS was conducted in negative electrospray ionization mode on an Agilent 6530 QToF-MS with an Agilent Jet Stream source. Source parameters were as follows: drying gas (N_2_) temperature 300 °C with a flow rate of 10 L/min; sheath gas (N_2_) temperature 400 °C with a flow rate of 12 L/min; nebulizer pressure 45 psig; capillary voltage 4750 V; nozzle voltage 1000 V; and fragmentor voltage 175 V. Reference ion solution containing 10 μM purine (*m/z* 119.0360 for [M-H]-) and 2.0 μM HP-0921 (*m/z* 966.0007 and 1033.9881 for [M-H]- and [M+HCOO]-, respectively) in 95:5 ACN:H2O was added post-column at 8 μl/min by an Agilent 1260 Infinity II isocratic pump. The QToF was tuned and calibrated in the 2 GHz extended dynamic range mode for the 100 to 3200 *m/z* range immediately prior to sample analysis. Full-scan spectra were collected at a rate of 2 Hz with a mass range of 100 to 3200 *m/z* and the data were saved in profile format.

HPLC-MS data acquisition and analysis were performed using MassHunter Workstation software (Agilent Technologies): Data Acquisition Workstation (v B.06.01, SP1) and Qualitative Analysis (v B.07.00, SP2). A database was constructed with formulas corresponding to combinations of one or more of the following carbohydrate residues: N-acetylhexosamine (HexNAc), hexose (Hex), deoxyhexose (presumed to be fucose; Fuc), N-acetylneuraminic acid (Neu5Ac), or N-glycolylneuraminic acid (Neu5Gc) plus or minus sulfonate (SO3) moieties. Putative O-glycans were all assigned unique, six digit “Glyco-codes”’; for example, “210,101_a” would correspond to a glycan of two HexNAc, one Hex, 0 Fuc, one Neu5Ac and 0 Neu5Gc, and one SO3 residues; the _a suffix denotes that this is the first of multiple HPLC-resolved isobars with the same formula. At a minimum, all combinations contained one reduced HexNAc. The database was used with the Find-by-Formula algorithm in MassHunter’s Qualitative Analysis to survey the data for formula matches within a ± 10.00 ppm mass accuracy limit and, when applicable, a ± 0.175 min retention time window of the same peak in other samples. Peak areas and retention times were processed further in Microsoft Excel. To account for differences in sample masses, peak areas for all detected glycans were normalized to the total glycan signals for each sample. Samples were analyzed in triplicate where possible and mean relative HPLC-MS peak areas were reported.

#### Capillary electrophoresis with laser-induced fluorescence detection (CE-LIF)

A non-reductive, ammonia-catalyzed β-elimination procedure was used to cleave O-glycans from mucins in their reducing forms (36, 98) which are initially trapped as glycosylamines. In brief, mucin samples, freeze-dried into 1.5 ml screw-capped centrifuge tubes, were suspended in 750 μl 28% ammonium hydroxide containing 100 mg/ml ammonium carbonate (Sigma); briefly (∼5 min) heating to 60 °C followed by sonication in a water bath sonicator (VWR) effectively dissolved all ammonium carbonate and thoroughly dispersed the mucin samples. Samples were heated for an additional 40 h after which they were cooled and concentrated on a SpeedVac centrifugal concentrator. The glycan and ammonium salt-containing residue was resuspended in 500 μl H_2_O, briefly centrifuged (10,000 *g*, 5 min, room temperature) to remove insoluble material, and subjected to ENVI-Carb SPE exactly as described for O-glycan alditols above. Note that the acidic SPE washes effectively convert glycans into their hemiacetal (reducing) forms. The salt- and protein-free O-glycans were then fluorogenically derivatized using 8-aminopyrene-1,3,6-trisulfonate (APTS) exactly as previously described (99). APTS-labelled glycans were electrophoretically resolved by CE-LIF on a ProteomeLab PA800 (Beckman-Coulter) using 50 cm × 50 μm internal diameter (44 cm to detector) fused silica capillary filled with a background electrolyte of 25 mM ammonium acetate, pH 4.75 and 0.1% polyethylene oxide (*i.e.* N-CHO buffer; SCIEX). Electrophoresis was carried out under reversed polarity (−30 kV) with samples being hydrodynamically injected (0.5 psi) at the cathode. Complete CE-LIF details have been previously described (99).

### Statistics

Statistical analyses were conducted within RStudio (R: 4.2.2) using R software (version 4.2.2; R Foundation for Statistical Computing). Barplots are shown as mean ± SD. Differences between groups were determined either through the Student’s *t* test if both groups passed the Shapiro-Wilks Normality test; or the Wilcoxon rank-sum test, if one or both groups failed the Shapiro-Wilks Normality test. Differences with *p* < 0.05 were considered significant.

## Ethics approval and consent to participate

Approval for the human analyses of the present study was given by the UBC Clinical Research Ethics Board (H21–3534,H16–03300, and H22–02645). Written informed consent to participate was obtained from all participants. No information given in this present study can be used to identify any study participant. The human studies abide by the principles articulated in the Declaration of Helsinki.

## Data availability

Data from this study are either included in this report or can be requested directly from the corresponding author. In addition, the mass spectrometry proteomics data have been deposited to the ProteomeXchange Consortium (100) *via* the PRIDE (101) partner repository with the dataset identifier PXD048242.

## Supporting information

This article contains Supporting information.

## Conflict of interest

The authors declare that they have no known competing financial interests or personal relationships that could have appeared to influence the work reported in this paper.

## Supporting information

Supplemental information

Fig2_related

Fig4_and_FigS5_related

Fig6_and_FigS7_related

Fig6_SCFA

FigS8_SCFA
