# Supplementary material for: Fecal-adherent mucus is a non-invasive source of primary human MUC2 for structural and functional characterization in health and disease
Source: J Biol Chem. 2024 Jan 24;300(3):105675. doi: 10.1016/j.jbc.2024.105675 (PMC10891339; doi:10.1016/j.jbc.2024.105675)
Supplement: Supplemental information [file mmc1.docx]

**SUPPORTING INFORMATION**

**Fecal-adherent mucus is a non-invasive source of primary human MUC2 for structural and functional characterization in health and disease**.

Noah Fancy^1^, Nitin^2^, Darrek Kniffen ^1^, Mackenzie Melvin^1^, Negin Kazemian^3^, Javad Sadeghi^3^, Clara A. Letef^1^, Leah D’Aloisio^1^, Amanda G. Copp^1^, Rain Inaba^1^, Geetkamal Hans^1^, Simin Jafaripour^1^, Natasha Haskey^1^, Maitreyi Raman^4^, Pirandis Daneshgar^2^, Kris Chadee^4^, Sanjoy Ghosh^1^, Deanna Gibson^1^, Sepideh Pakpour^3^, Wesley Zandberg^2^, Kirk Bergstrom*^1^

^1^Biology, ^2^Chemistry, ^3^School of Engineering, University of British Columbia - Okanagan, Kelowna, ^4^Cumming School of Medicine, University of Calgary, Calgary, Canada

*Corresponding author

List of Materials included in this document:

- Figure S1 – S8.

List of Materials included online:

- Fig2_related.xlsx: Figure 2 proteomics datasets
- Fig4_and FigS5_related.xlsx: Figure 4 and Figure S5 glycomics datasets
- Fig6_related.xlsx: Figure 6 and Figure S7 glycomics datasets
- Fig6_SCFA.xlsx : Figure 6 metabolomics data
- FigS8_SCFA.xlsx: Figure S8 metabolomics data


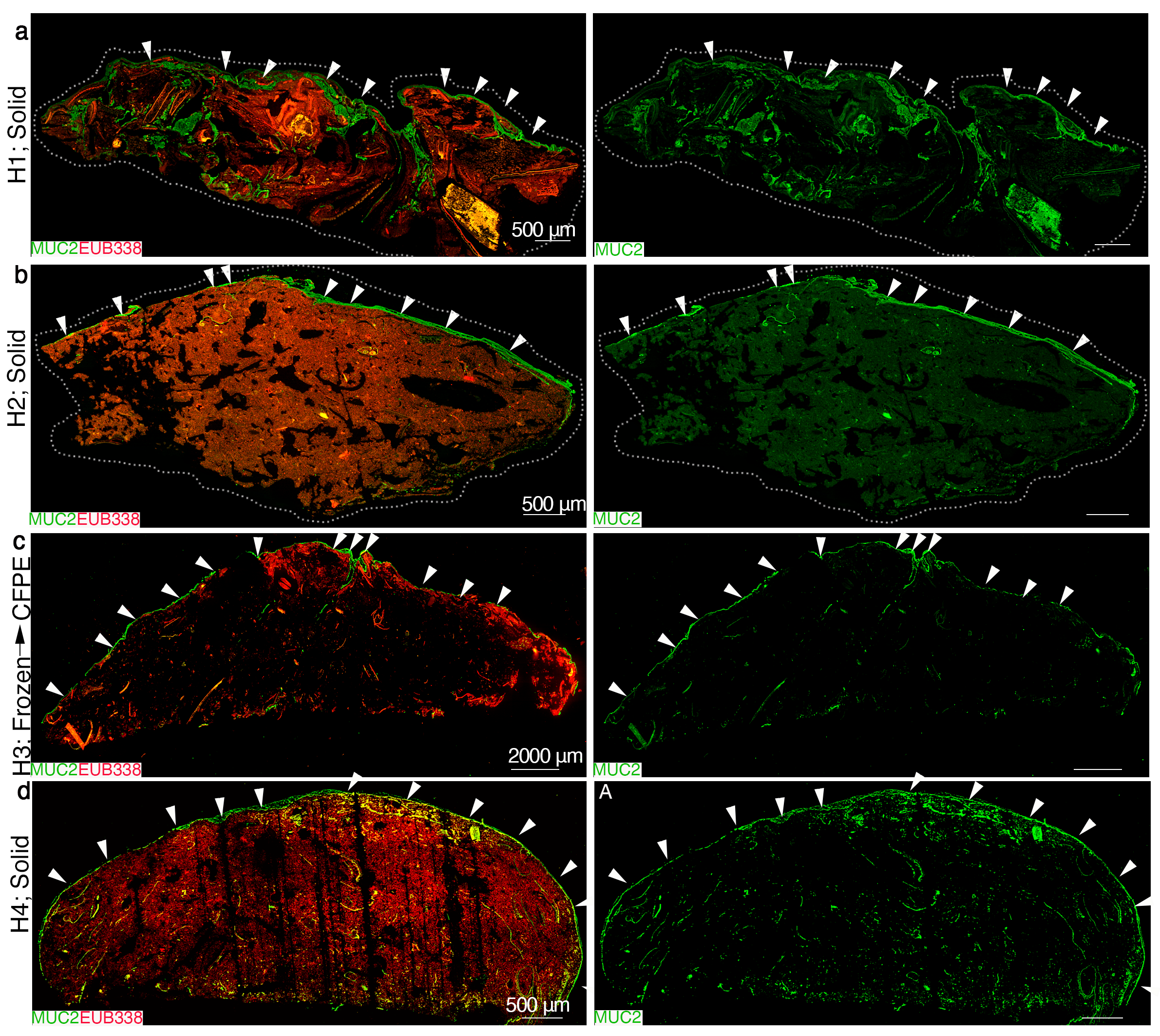


**Figure S1. Visualization of the mucus layer on fecal sections from different individuals.**  MUC2:FISH-stained tiled image of CFPE healthy human fecal cross sections from a South Asian female (**a**), South Asian male (**b**), Caucasian female (**c**) and Caucasian male (**d**). The designations (H1, H2, H3, H4) refer to those quantified in Fig 1. Dashed lines delineate the boundary of the image.


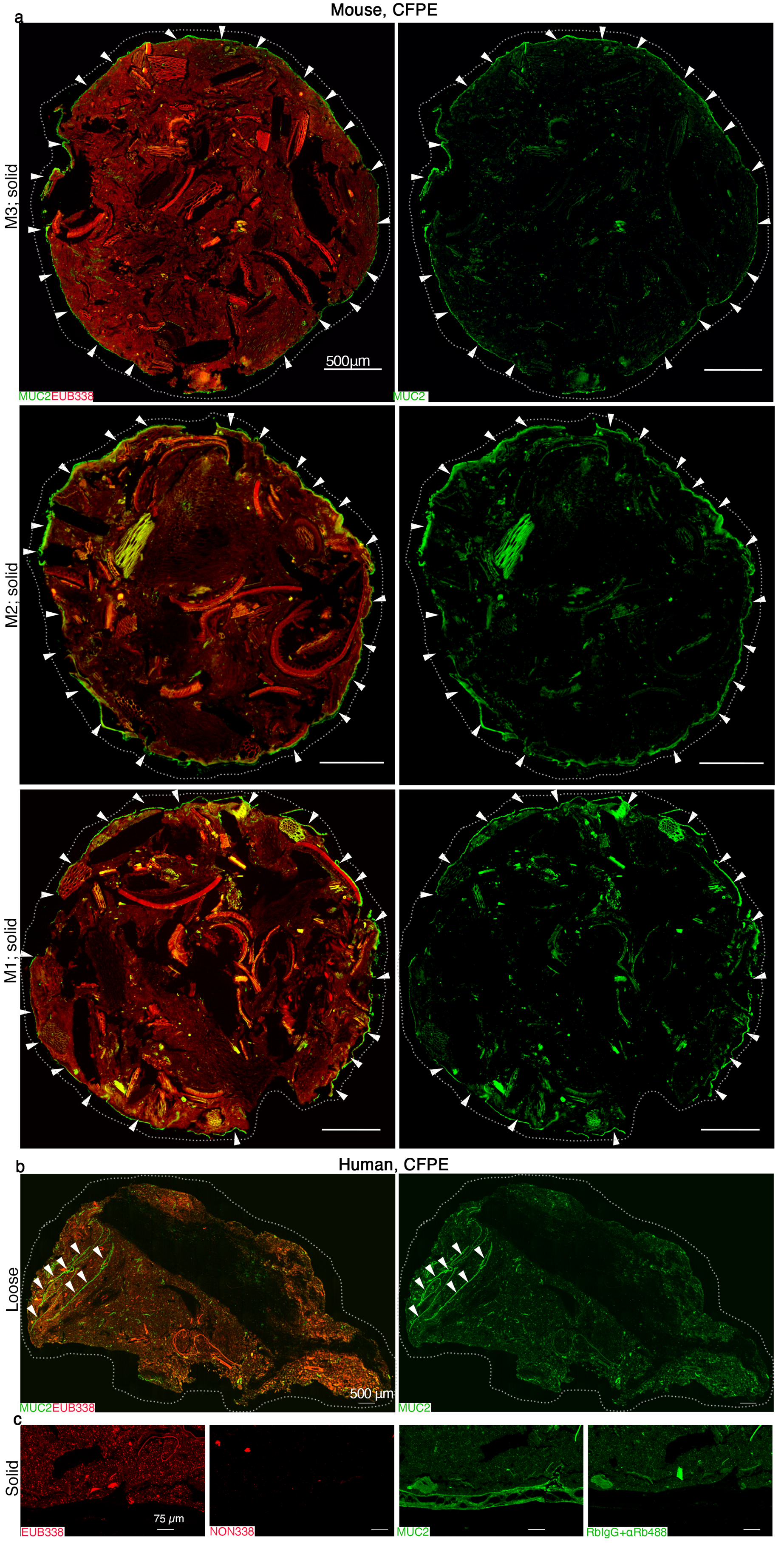


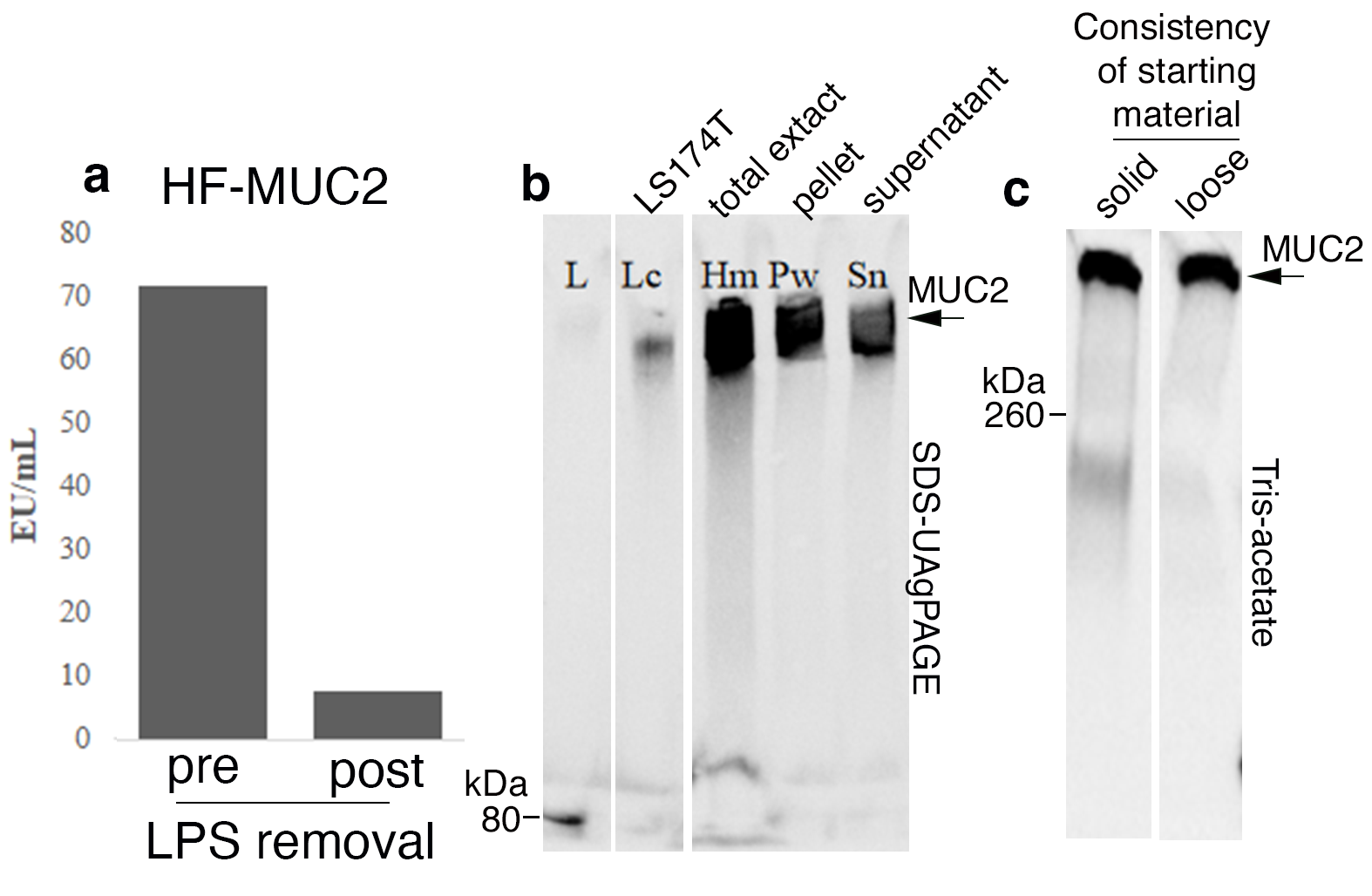


**Figure S2. Visualization and validation of the mucus layer on fecal sections from individual mice and human stools. a**. MUC2:FISH-stained tiled image of CFPE healthy mouse fecal cross sections from female C57BL/6 mice. Arrowheads point to continuous MUC2 strands distributed around the fecal mass. The designations (M1, M2, M3) refer to those quantified in Fig 1. **b**. Loose CFPE fecal section from a Caucasian male. Arrowheads point to continuous MUC2 strands distributed around or within the fecal mass, depending on its consistency. **c**. High magnification image of a human fecal section dually stained for MUC2/EUB338 or Rabbit IgG isotype control and nonsense probe NON338 to confirm specificity of the MUC2 antibody and FISH probes. Dashed lines delineate the boundary of the image.

**Figure S3. Characterization of LPS levels in extracted MUC2 and assessment of extracted MUC2 in loose feces**. **a**. Endotoxin (LPS) quantification in fecal MUC2 before and after treatment with an endotoxin removal column. **b**. Western blot of different fractions of the purified human fecal mucin lysate from our protocol. The dialyzed lysate contains a solid precipitate. Lane 1, a mix (“Hm”) of insoluble and soluble material; lane 2, solid material (pellet, “Pw”) only; lane 3, supernatant (“Sn”) only. The solid material was determined to be a composite of LPS and MUC2. **c.** Western blot of extracted MUC2 from solid and loose fecal material, separated by a tris-acetate gradient gel. White space between lines indicates lanes that were not adjacent on the original gel, but were cropped to remove irrelevant data and re-organized for preferred order.


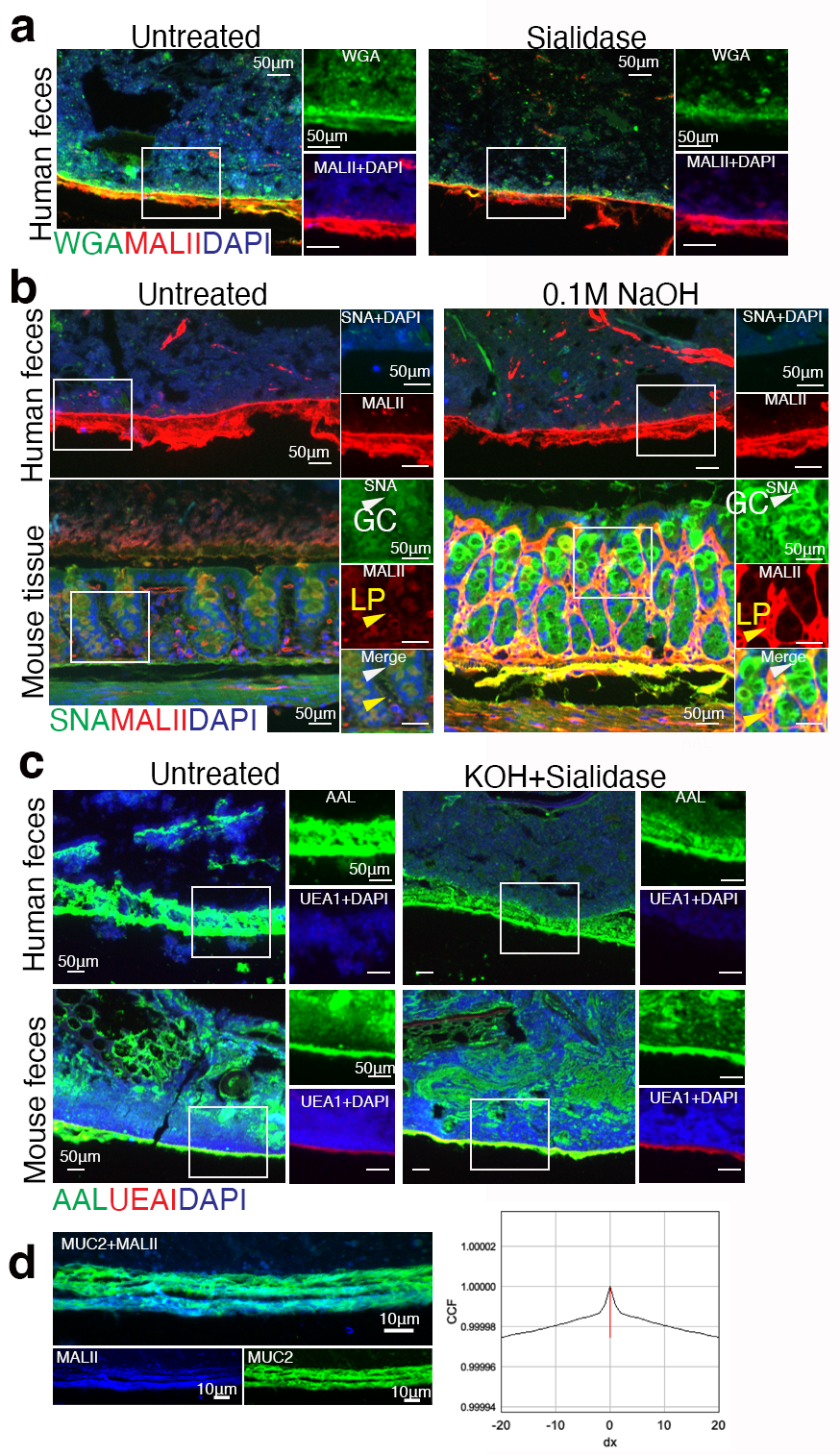


.

**Figure S4. In situ biochemical treatments to identify O-glycan types**. **a**. Sialidase treatments on human fecal sections: Broad-spectrum Sialidase (α2-3,6,8) reduced WGA staining, but did not impact MALII staining, suggesting sialyation is present in human MUC2, but MALII is unlikely recognizing α2,3 linked Sia but rather sulfated variants. **b**. Saponification of CFPE sections to determine if O-acetyl modifications can explain the lack of SNA staining. Mouse tissues are a positive control for SNA staining, and reveal that saponification unmasks SNA epitopes in mouse colon goblet cells and lamina propria of mouse proximal colon, but not in human MUC2. MALII epitopes were unchanged in human sections, but unmasked only in mouse colon lamina propria. **c**. AAL and UEA1 staining following saponification and neuraminidase treatment to determine if sialylation is blocking UEA1 binding. Saponification is first used to remove O-acetylation which can block neuraminidase activities, and neuraminidase should remove Sia’s blocking UEA1 binding. UEA1 signal was unaffected by the treatment. **d.** Confocal and colocalization analysis of MALII:MUC2 labeling. *Right*: Van Steesels Cross Correlation Function (CCF) plot showing perfect correlation of the lectin and MUC2 signal, demonstrated by the peak at centre of the plot, indicating colocalization.


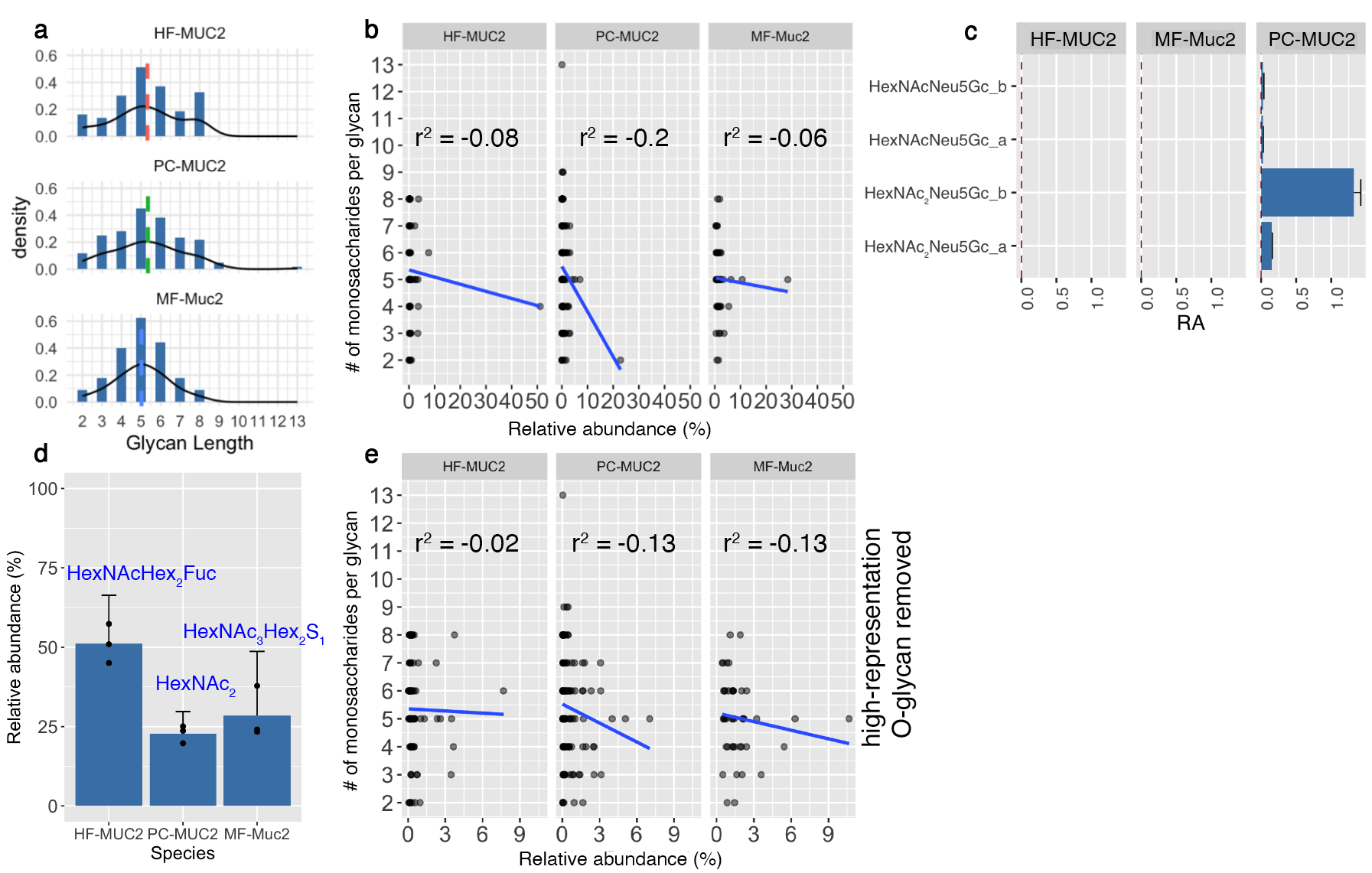


**Figure S5. Characterization of overall O-glycosylation status**. **a**. Histogram showing levels of glycans of different specific lengths (i.e. numbers of monosaccharides) for each sample and species. The plot is overlaid with a density curve to describe shape of the data. Dotted vertical line is the mean glycan length. **b.** Scatterplots of glycan lengths vs. relative abundance of each glycan. Blue line represents curve fit for linear regression model. **c**. Mean relative abundance of glycans containing Neu5Gc. **d**. Barplot of means (+/- 95% confidence intervals) of unique glycans that were disproportionately high (i.e. > 20% contribution to relative abundance). Each point is a technical replicate (independent run). The glycan compositions are given above. **e**. Same as “b” but with the disproportionately high glycans from “c” removed from the analysis.


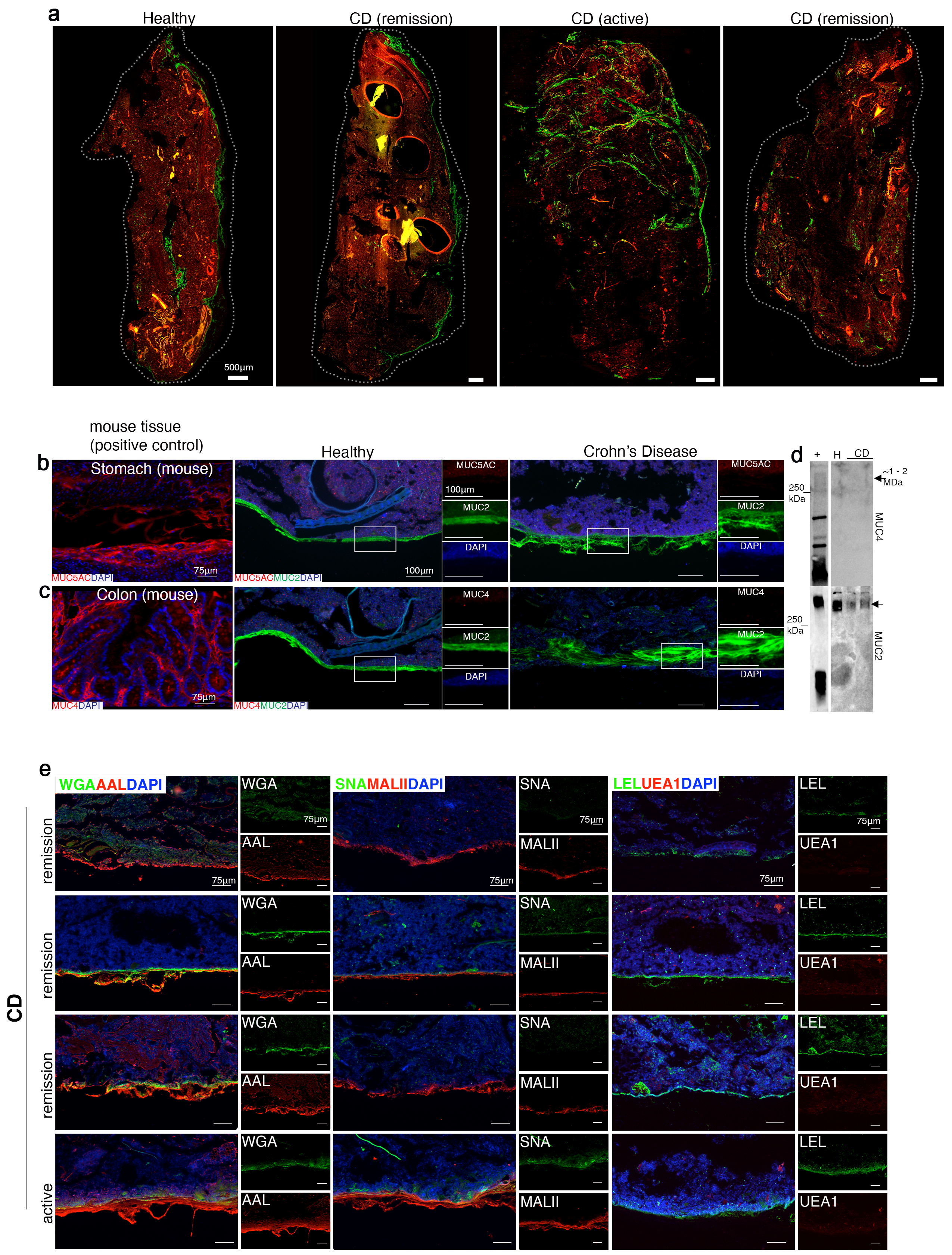


**Figure S6.** **In-situ investigation of Crohn’s Disease fecal MUC2. a**. MUC2:FISH-stained tiled image of CFPE healthy human fecal cross sections. Dashed lines delineate the boundary of the image. **b**. Immunostaining of mouse anti-MUC5AC IgG (red) in mouse stomach (positive control) and human fecal sections, the latter co-stained with MUC2 (green). Magnified split images of boxed regions are shown on the right. **c**. Immunostaining as above except using mouse anti-MUC4 IgG (red). **d**. Western blot for MUC4 on mouse colon lysate (+ control) and HF-MUC2. MUC2 immunoblots are shown below as loading controls. **e**. Epifluorescent staining for various lectin combinations in CFPE CD fecal sections. Split images are shown to the right of each panel.


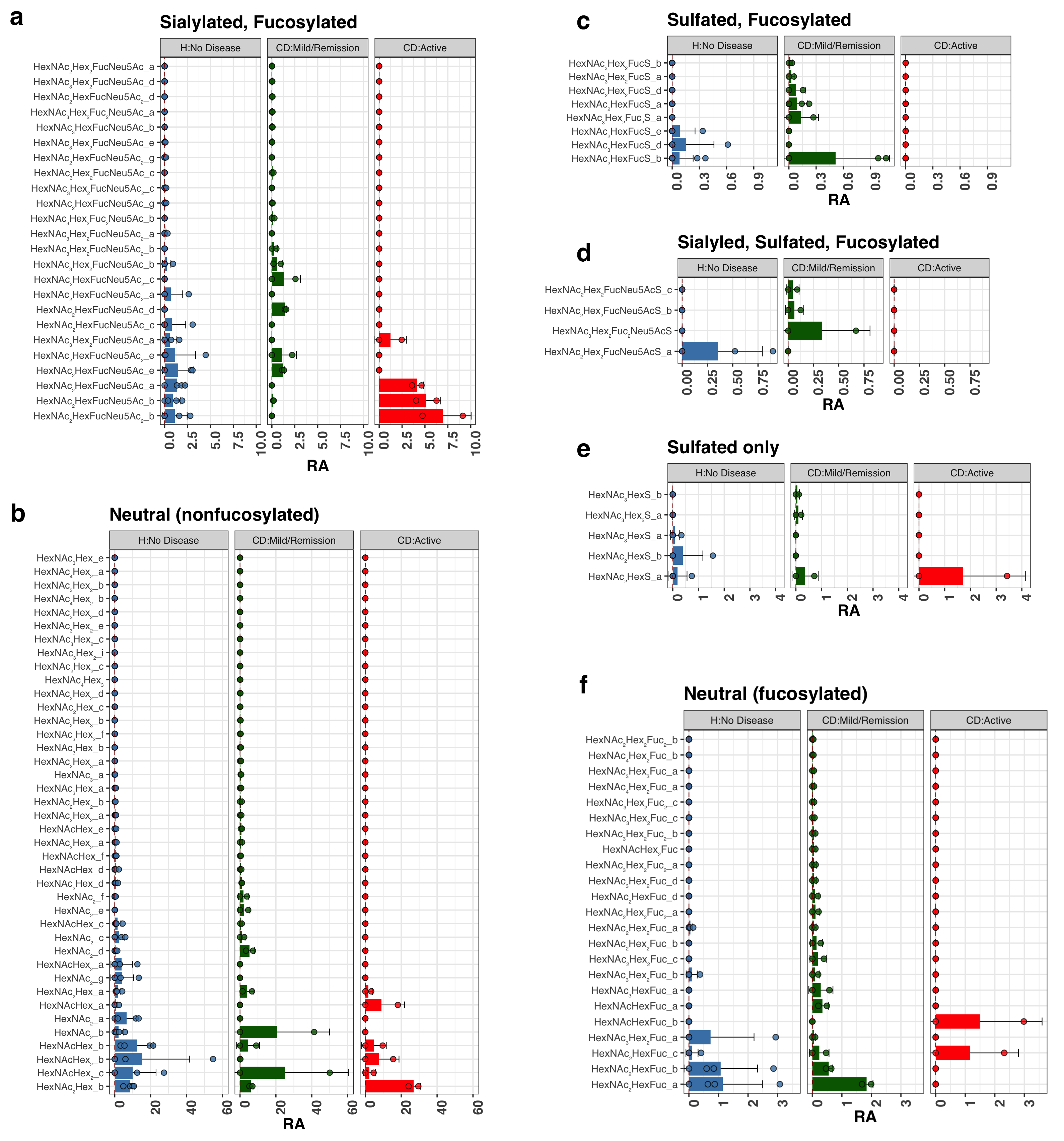


**Figure S7.** **Crohn’s Disease fecal MUC2 glycosylation a. - f**. Barplots showing mean +/- SD (n = 4 healthy, 4 CD (2 mild, 2 active)) of the relative abundances of individual glycans categorized according to subclass.


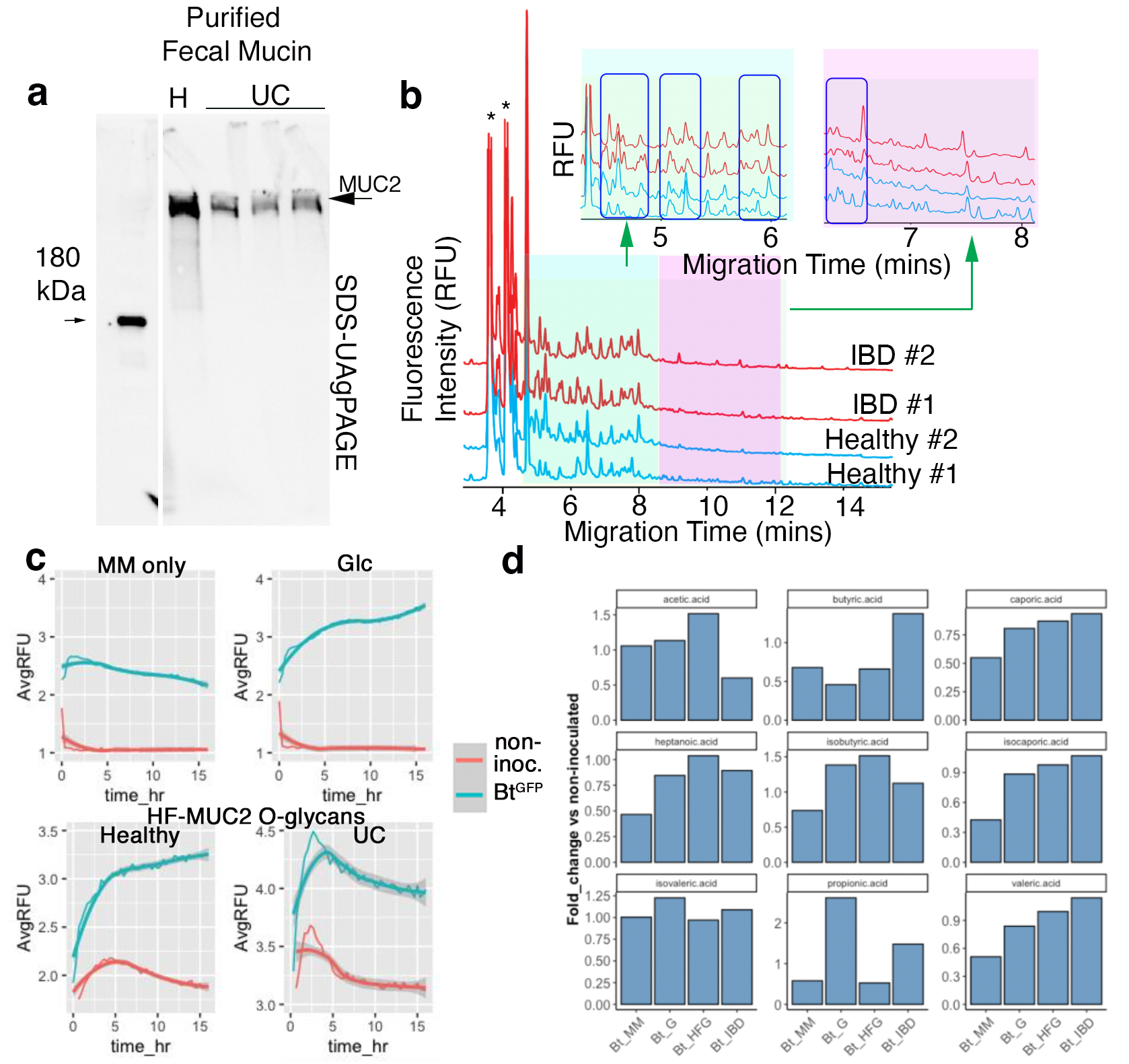


**Figure S8.** **Comparison of human fecal MUC2 from healthy and Ulcerative Colitis samples**. **a**. Western blot of MUC2 purified from feces of healthy vs. Ulcerative Colitis (UC) patients. **b**. Capillary electrophoresis-laser induced fluorescence showing glycoprofiles of healthy vs. UC patients. Each peak is an individual glycan. Upper right: zoomed-in regions of shaded portions in the base chromatogram. Boxes show peaks of interest. **c**. Growth curves of *B. theta*-GFP (*Bt^GFP^*) in the presence of glycans from healthy vs. UC patients. The experiment was performed twice. **d.** Bar plot of short-chain fatty acid profiles taken from spent supernatants of *Bt*^GFP^ grown in minimal media with or without human MUC2 O-glycans. Results show fold change in SCFA produced in inoculated media vs. non-inoculated media. Bt-MM: Minimal media without any carbon source; Bt_G = Minimal media supplemented with Glucose (Positive control for growth in minimal media; Bt_HFG = Minimal media + healthy human fecal MUC2 O-glycans. Bt_IBD = Minimal media supplemented with UC fecal MUC2 O-glycans. Glycans were pooled to provide enough for the assay, therefore no error bars are shown for each group.
